# The Triple Combination Phentermine Plus 5-HTP/Carbidopa Leads to Greater Weight Loss, With Fewer Psychomotor Side Effects Than Each Drug Alone

**DOI:** 10.3389/fphar.2019.01327

**Published:** 2019-11-06

**Authors:** Claudia I. Perez, B. Kalyanasundar, Mario G. Moreno, Ranier Gutierrez

**Affiliations:** Laboratory of Neurobiology of Appetite, Department of Pharmacology, CINVESTAV, Mexico City, Mexico

**Keywords:** 5-hydroxytryptophan/carbidopa, phentermine, antiobesity drugs, appetite suppressants, obesity

## Abstract

Obesity has become a serious public health problem. Although diet, surgery, and exercise are the primary treatments for obesity, these activities are often supplemented using appetite suppressants. A previous study reported that obesity specialists frequently prescribed a new drug combination for its treatment that includes phentermine (Phen; dopaminergic appetite suppressant), a serotonin (5-HT) precursor 5-hydroxytryptophan (5-HTP; an appetite suppressant that increases the 5-HT concentration), and carbidopa (CB; peripheral blocker of conversion of 5-HTP to 5-HT). Despite its widespread use, there is neither a preclinical study confirming the drug efficacy nor studies of its effects on the brain. To fill this gap, in rats for seven consecutive days, we administered Phen intraperitoneally at different doses either alone or in combination with a fixed dose of 5-HTP/CB. In a different group, we infused drugs *via* an intraperitoneal catheter while extracellular-recordings were performed in the nucleus accumbens shell (NAcSh), a brain region with dopamine-releasing effects that is involved in the action of appetite suppressants. We found that the triple-drug combination leads to greater weight-loss than each drug alone. Moreover, and as the treatment progresses, the triple drug combination partially reversed psychomotor side-effects induced by Phen. Electrophysiological results revealed that Phen alone evoked a net inhibitory imbalance in NAcSh population activity that correlated with the onset of psychomotor effects. In addition, and unlike the greater weight loss, the addition of 5-HTP/CB did not alter the Phen-evoked inhibitory imbalance in NAcSh responses. Subsequent experiments shed light on the underlying mechanism. That is the majority of NAcSh neurons modulated by 5-HTP/CB were suppressed by Phen. Notably, and despite acting *via* a different mechanism of action (DA for Phen vs. 5-HT for 5-HTP/CB), both drugs recruited largely overlapping NAcSh neuronal ensembles. These data suggest that the neural correlates of the greater weight loss could be located outside the NAcSh, in other brain circuits. Furthermore, we conclude that Phen + 5-HTP/CB is a potential treatment for overweight and obesity.

## Introduction

One common treatment of the obesity epidemic involves the use of drugs (Hampp et al., 2013; Valsamakis et al., 2017; Srivastava and Apovian, 2018). One of the most widely prescribed of these drugs for humans in the USA is Phentermine (Phen), which is an appetite suppressant currently approved for the short-term treatment of obesity (Hendricks et al., 2009; Hendricks et al., 2011; Hendricks et al., 2014; Yanovski and Yanovski, 2014). Studies in rodents have also shown that repeated administration of Phen induces body weight loss, decreases food intake, and several psychomotor side effects such as locomotion and stereotypy (Kalyanasundar et al., 2015). It has been shown that Phen promotes norepinephrine (NE) release and inhibits dopamine (DA) reuptake, presumably *via* a substrate-type releaser action that increases extracellular DA efflux by a diffusion exchange process involving the dopamine transporter (DAT) (Balcioglu and Wurtman, 1998; Baumann et al., 2000). In contrast, 5-hydroxytryptophan (5-HTP) is a serotonin precursor, while carbidopa (CB) is a peripheral decarboxylase inhibitor that blocks the conversion of 5-HTP to serotonin (5-hydroxytryptamine, 5-HT) at the periphery. Thus, the combination of 5-HTP/CB increases 5-HT levels on the brain, reducing peripheral side effects (Turner et al., 2006; Beakley et al., 2015). In rodents and humans, 5-HTP suppresses food intake and induces body weight loss (Amer et al., 2004; Halford et al., 2005).

Previously, we tested Phen and two other like compounds (diethylpropion and bupropion) that affect DA in the nucleus accumbens shell (NAcSh) for effects on body weight-loss, food suppression, and evoked neuronal responses (Kalyanasundar et al., 2015). Here, we have extended these studies to 5-HTP. In this regard, Hendricks et al. (2009) reported that obesity specialists widely prescribe Phen plus 5-HTP/CB surpassing other combinations (e.g., caffeine + ephedrine or sibutramine + orlistat) to reduce feeding. Nevertheless, it is not well understood how these three drugs interact with each other to induce greater weight loss. What is clear, is that, in addition to its effects on weight loss, DA releasers’ drugs are potent locomotor stimulants (e.g., amphetamine, diethylpropion, and Phen), while drugs that preferentially release 5-HT (e.g., fenfluramine and chlorphentermine) do not stimulate locomotion (Baumann et al., 2000; Rothman et al., 2006). Moreover, 5-HT can antagonize locomotor stimulation produced by DA releasing agents (Rothman and Baumann, 2006). Specifically, the administration of fenfluramine (5-HT releaser) reduces the psychomotor effects of Phen (Baumann et al., 2000). In rodents, similar results are found with the combination of d-amphetamine (a prototypical DA releaser) and 5-HTP plus benserazide (just like CB, another peripheral decarboxylase inhibitor) (Baumann et al., 2011). Thus, the interaction DA/5-HT might be useful from a therapeutic standpoint. However, it remains to be demonstrated whether this is the case for the combination of Phen + 5-HTP/CB. Likewise, there is little, if any, clinical support about this combination. To the best of our knowledge, there is only one pilot study in 91 human patients, evaluating the efficacy and safety of the combination Phen + 5-HTP/CB on weight loss, which produced promising results (Rothman, 2010). However, the Food and Drug Administration has not approved its use in the USA. Moreover, it has not been performed a randomized placebo-controlled clinical trial. In conclusion, the efficacy of the triple-drug combination remains to be tested in humans, as well as in preclinical studies in rodents. To address this paucity of information, here, we will evaluate, in rats, the efficacy of the triple-drug combination for weight loss and characterize the neuronal responses evoked in the NAcSh.

The NAcSh is a potential target of several appetite suppressants (Nowend et al., 2001; Kelley, 2004; Green et al., 2011). The NAcSh is the main region of the striatum that projects to the lateral hypothalamus, allowing it to control feeding (O’Connor et al., 2015; Prado et al., 2016). The NAcSh is composed of 95% of medium spiny neurons (MSNs) that either express D1 or D2-like receptors. Since, MSNs exhibit low spontaneous activity, its firing reflects the balance and integration of three main neurochemical inputs (Klawonn and Malenka, 2019): the glutamatergic inputs from cortex (among other brain regions), the mesolimbic DA from the ventral tegmental area, and serotonin input from 5-HT containing neurons from dorsal raphe nuclei (Hensler, 2006; Klawonn and Malenka, 2019). Its anatomical connections indicate that the NAcSh should be a potential pharmacological target of appetite suppressants (Rothman and Baumann, 2006; Kalyanasundar et al., 2015). Recently, extracellular recordings in this brain region revealed that Phen and other appetite suppressants of this class, including diethylpropion and bupropion, evoke a global inhibitory imbalance in NAcSh responses (Kalyanasundar et al., 2015). Furthermore, blockade of D1 and D2-like DA receptors in the NAcSh markedly reverse the effects of appetite suppressants upon locomotion, food intake, weight loss, and restored neuronal responses, thus pointing to an important role for D1/D2-like receptors in the mechanism of action of this class of anorexigenic compounds (Kalyanasundar et al., 2015). Moreover, DA and serotonin in the NAcSh have been associated with feeding behavior (Pothos et al., 1995; Xu et al., 2017; van Galen et al., 2018), but how these neurotransmitters influence NAcSh ensemble activity, however, is less understood.

Here, we used behavioral measurements and extracellular recordings in the NAcSh of awake behaving animal, while Phen + 5-HTP/CB were administered either as monotherapy or in combination. Our data support Phen plus 5-HTP/CB as a triple-drug combination that shows potential as a treatment of overweight and obesity.

## Materials and Methods

### Animals

Male Sprague-Dawley rats (n = 150, Harlan Mexico) of approximately 81 days post-natal (∼300–330 g) were used for all experiments. Animals were housed individually. Rats had *ad libitum* access to food and water at homecages except when they were in the open-field arena or during extracellular recordings (see below for details). Rats were maintained at ∼21°C and in a 12:12-h light-dark cycle, in which lights were on at 0600 and off at 1800 h. All procedures were approved by the CINVESTAV institutional animal care and use committee.

### Drugs

Phen, 5-HTP, and CB were kindly donated by Productos Medix (Mexico). Phen was dissolved in physiological saline (Phentermine HCl in powder), CB was suspended in carboxymethyl cellulose (0.5%, Sigma-Aldrich) and saline in a proportion 1:4 (hereafter referred to as the vehicle). 5-HTP was dissolved in saline at ∼40°C and maintained at warm temperature (for no more than 40 min) until injection (O’Neil, 2001). All drugs were prepared and administered daily. For behavioral experiments, drugs were injected intraperitoneally, whereas for electrophysiological recordings, to reduce stress due to handling the drugs were infused *via* an intraperitoneal catheter (*see Methods below*).

### Behavioral Procedures

#### Dose-Response Curves of Phen and 5-HTP/CB on Weight-Loss

To determine whether Phen and 5-HTP/CB affect body weight, we measured body weight and 24 h food intake daily. For all groups, rats received injections for seven consecutive days during the light phase between 1430 and 1600 h.

Since Hirai and Nakajima (1979) showed that the highest dose of 5-HTP (LD_50_ 400 mg/kg) could cause kidney damage, we always administered a fixed dose of CB (75 mg/kg), 30 min before infusion of 5-HTP. Moreover, in a pilot study, we found that CB75 alone did not induce weight loss, but relative to the saline group, it prevented rats from gaining weight (**Supplementary Figure 1**). Thus, we administrated different doses of 5-HTP/CB using 27 rats that were randomly assigned to 6 groups: Vehicle (n = 5), 5-HTP1/CB (n = 4), 5-HTP12.5/CB (n = 5), 5-HTP25/CB (n = 4), 5-HTP50/CB (n = 5), and 5-HTP100/CB (n = 4), where numbers indicate the dose of 1, 12.5, 25, 50, and 100 mg/kg of 5-HTP, respectively.

For the dose-response curve of Phen, we assigned 30 rats to 6 groups (n = 5): saline, Phen1, Phen3, Phen10, Phen30, and Phen45, where numbers indicate doses of 1, 3, 10, 30, and 45 mg/kg, respectively. In all experiments, rats were given in their homecages 100 g allotment of standard rat chow per day (Purina, Mexico) and *ad libitum* tap water.

#### Effect of 5-HTP/CB or Phen Alone and in Combination on Weight Loss and Food Intake

To evaluate if Phen + 5-HTP/CB combination induces greater weight loss than each drug alone, we combined several doses of Phen (hereafter Phen10, Phen15, and Phen20, respectively) with a fixed dose of 5-HTP/CB (31 mg/kg). A total of 63 rats were randomly assigned to eight groups: vehicle (n = 8), 5-HTP/CB (n = 8), Phen10 (n = 5), Phen15 (n = 8), Phen20 (n = 8), Phen10 + 5-HTP/CB (n = 5), Phen15 + 5-HTP/CB (n = 11), and Phen20 + 5-HTP/CB (n = 10). As noted, we always administered a pretreatment injection of CB 30 min before 5-HTP, followed by Phen. Right after the last injection, each animal was placed in an open field arena, for 90 min in order to measure locomotion activity.

#### Open Field Arena and Locomotor Activity

For behavioral experiments, locomotor activity was measured in an open field arena (40 cm length x 40 cm width x 40 cm height) coupled with a camera (in top view position). The animal’s position in *x* and *y* coordinates was tracked using Ethovision XT10 (Noldus Information Technology, Netherlands). One single video could simultaneously track up to four open fields. The forward locomotion was tracked using the rat’s center mass method and plotted as total distance traveled (cm) during 90 min from the onset of injection. One video was lost on the 3^rd^ day of treatment, and thus, data from four animals were not included in this analysis (one rat for Phen20 and 5-HTP/CB; and two rats for Phen20 + 5-HTP/CB groups).

#### Measurements of Stereotypy

The stereotypy, in the form of head weavings, was hand-scored using the Manual Scoring Setting on the Ethovision XT10 software. We focused on head weavings (obvious lateral swaying movement of the head) since this is the most prominent stereotypy induced by Phen (Kalyanasundar et al., 2015). Stereotypy was measured from a 5 min video, around ∼1 h after onset of Phen or 5-HTP/CB injection. This is because Phen stimulates its maximum psychomotor effects around this time (Kalyanasundar et al., 2015). Four behavioral states were measured: head weaving stereotypy, moving, quiet awake, and sleep. In stereotypy, while immobile animals exhibit head weavings. We acknowledge that head weavings could occur during other physiological behaviors such as walking, rearing, and exploration. However, head weavings during these behaviors were not counted as stereotypy. In moving, the animal was moving in the arena or rearing. In quiet awake state, the animal was immobile with their eyes open but without head weavings. While sleeping, the animals were lying immobile, with their eyes closed and without head weaving. The results were displayed as the percentage of the accumulative time spent in each behavioral state.

### Electrophysiology

*Surgery*. Surgical procedures for electrode implantation targeting the NAcSh has been previously described in detail (Kalyanasundar et al., 2015). Briefly, animals were anesthetized using an intraperitoneal injection of xylazine (8 mg/kg) and ketamine (80 mg/kg). A homemade electrode array, composed of 16 tungsten wires (35 µm diameter) 4x4 (1 mm^2^), was unilaterally implanted in the right hemisphere (from Bregma at coordinates AP +1.4 mm, ML -1 mm, and DV -7.5 mm). For ground, we used a stainless-steel screw soldered to a silver wire that was inserted above the cerebellum. For intraperitoneal catheter implantation, we followed the protocol described by (Rüttimann et al., 2009; Boudreau et al., 2010). Briefly, the catheter was sterilized in 70% alcohol overnight. First, the hair, on the lower left quadrant of the animal’s abdomen and dorsal neck areas, was shaved, and the skin cleaned with an iodine solution. We used silicone tubing as a catheter (15 cm length; Silastic laboratory tubing, Dow Corning, Midland, MI, CAT. No. 508-004). The catheter was introduced in a midline incision (1 cm length) into the abdominal cavity and attached to the abdominal wall *via* a rubber band brace, 1 cm from catheter’s tip using silk suture (USP 3-0, Atramat, Mexico). We passed the opposite tip of the catheter subcutaneously until it exited the dorsal neck incision. Ketoprofen (5 mg/kg, Ketofen 1%) and Enrofloxacin (10 mg/kg, Baytril 5%) were administered intraperitoneally for three consecutive days after surgery. To maintain patency, catheters were flushed daily with saline (0.9%). Rats were allowed seven days for recovery. After this recovery period, for three more days, they were habituated to the operant box. We infused saline or the drugs using a silicone tube connected to the rat’s catheter (∼45 cm length Silastic laboratory tubing, Dow Corning), *via* a manually operated syringe located outside of the behavioral box.

#### Extracellular Recordings of NAcSh

Electrophysiological experiments were performed in an operant behavioral box (31 cm x 24 cm x 21 cm Med Associates) enclosed in a ventilated and sound-attenuating chamber. Recordings took place at 1100 h daily, and they were coupled to a video camera (in top view position). Recordings were performed using a multichannel acquisition processor (Plexon, Dallas, TX). Voltage signals were sampled at 40 kHz and digitalized at 12 bits resolution, and action potentials were band-passed filter (154 Hz–8.8 kHz). Action potentials with a signal-to-noise ratio larger than 3:1 were analyzed and identified online by mean of voltage-time threshold windows and a three-principal component contour template algorithm (Gutierrez et al., 2010). Spikes were sorted using Off-line Sorter software (Plexon inc). During multichannel recordings, the position of the rat’s center of mass was tracked offline using Ethovision software (Noldus Information Technology, Netherlands). We only included in the analysis videos in which locomotion could be decoded. We note that, during the recording sessions, the stereotypy behavior could not be measured since the cable limited visibility of the head.

*Modulation of NAcSh during the injection of Phen, or 5-HTP/CB, or Phen + 5-HTP/CB*. We used 10 naïve rats to measure NAcSh neuronal activity [Phen (n = 3), 5-HTP/CB (n = 3), and Phen + 5-HTP/CB (n = 4)]. Each recording session lasted 3 h, and it was divided into four epochs: Baseline (period without drug infusion, BL: 0–59 min), Saline (Sal: 60–89 min), Carbidopa (CB: 90–119 min; when 5-HTP was infused), or Saline (90–119 min; when Phen alone was injected), and Phen followed by infusion of 5-HTP (120–180 min). Before each recording session, the body weight and 24 h food intake were measured during seven consecutive days. We decided to use the ED_50_ dose of Phen (15 mg/kg) and ED_45_ for 5-HTP (31 mg/kg). Phen doses were chosen because they are the most commonly used (Baumann et al., 2000; Roth et al., 2008; Kalyanasundar et al., 2015), whereas the selected 5-HTP dose is known to reverse psychomotor effects induced by d-amphetamine (Baumann et al., 2011).

*Modulation of NAcSh neurons, in the same animal, during the injection of 5-HTP followed by Phen.* To further characterize how 5-HTP and Phen modulate the same NAcSh neuron activity, we used five additional male rats implanted with an electrode array targeting this brain region, each session lasted 3 h, and each drug was infused following the next order: BL (0–29 min), Saline (30–59 min), CB (60–89 min), 5-HTP (90–134 min), and then Phen (135–180 min). Since Phen evoked the largest NAcSh modulation, it was administered at the end.

*Histology.* Rats were injected with an overdose of sodium pentobarbital (150 mg/kg) and perfused with PBS intracardially, followed by paraformaldehyde at 4%. The brain was removed and placed in a 10% sucrose solution for 24 h, followed by sequential increases in sucrose concentration until reaching 30% wt./v in 72 h. The brain was sliced (50 µm) and stained with cresyl violet to establish the location of the electrodes’ tip.

#### Quantification and Statistical Analysis

*Behavioral analysis.* Data are mean ± sem. Statistical differences between groups were analyzed using repeated measures ANOVA (RM ANOVA) followed by a Tukey *post hoc*, using GraphPad Prism 6 and StatView software.

*The dose-response curve of Phen or 5-HTP/CB*. We calculated the effective dose using the equation *f* = min + ((max-min)/[1 + (EC50/X)^Hill slope]), where *f* is the expected response to a given dose (X), min and max are the lowest and highest weight loss, and the EC50 is the dose at which 50% of the subjects are expected to show the desired response using the program of Gadagkar and Call (2015).

#### Electrophysiological Analysis

We used custom made scripts in Matlab (The MathWorks Inc., Natick, MA) to analyzed neuronal responses.

Neurons that increase or decrease their firing rate after Phen, or 5-HTP/CB, or Phen + 5-HTP/CB. Neuronal responses were classified as decreasing or increasing firing rates relative to baseline (BL). After treatment (Phen, or 5-HTP, or Phen + 5-HTP), significant changes in firing rates were identified using a Kruskal Wallis test with an α < 0.05. Neurons with increasing responses during treatment relative to BL were named “Increase,” whereas neurons with decreased activity “Decrease.” Neurons with no significant modulation were not-modulated “NoM.” We used a chi-square test to assess differences in the proportion of neurons recruited by each treatment.

## Results

### Behavioral Results

#### Dose-Response Curve of 5-HTP/CB on Weight-Loss

**Figure 1:**
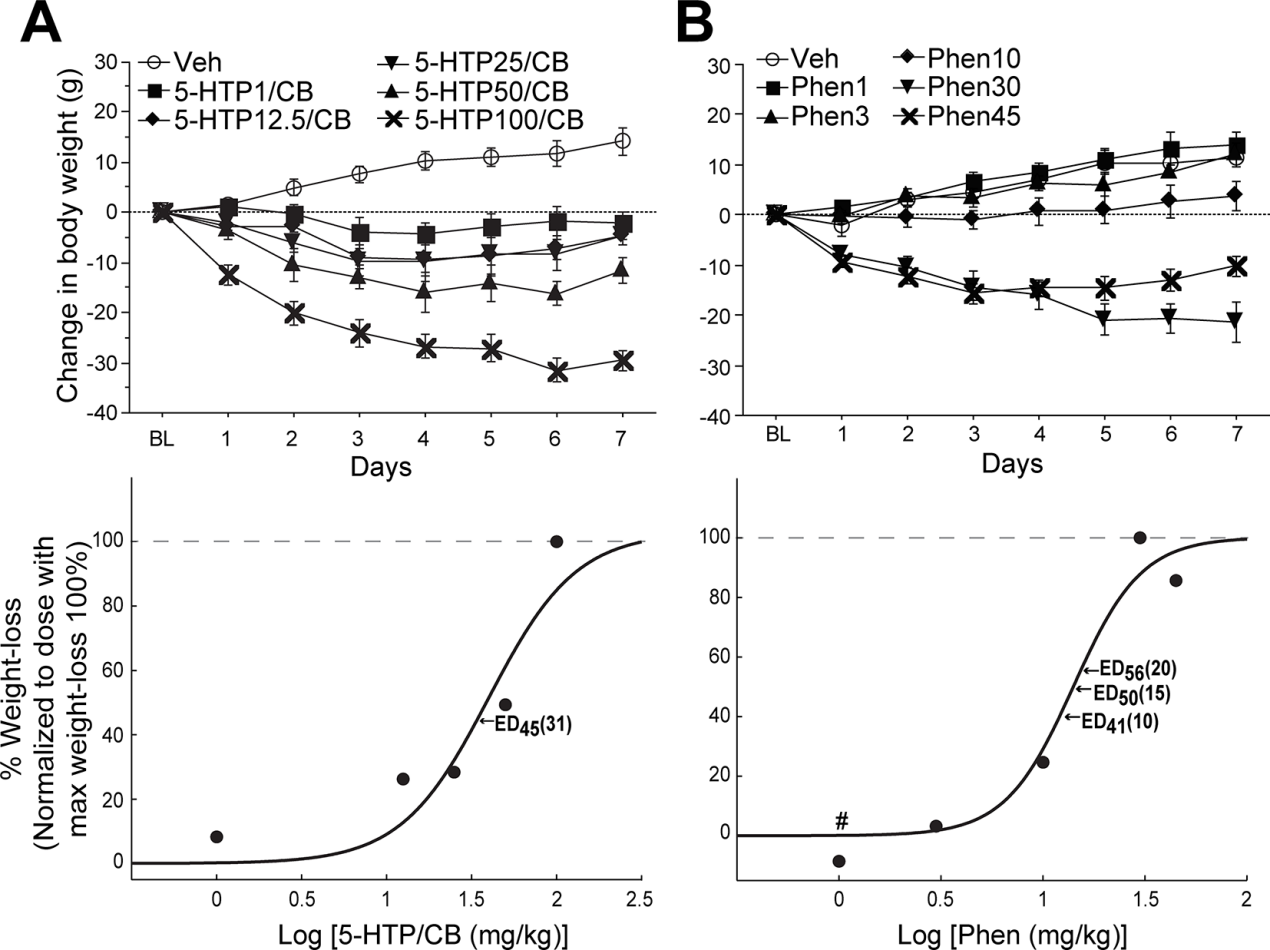
Dose-response curve of 5-hydroxytryptophan (5-HTP)/carbidopa (CB) or phentermine (Phen) alone in rats. **(A)** *Upper panel:* the change in body weight after seven days of intraperitoneal injection of vehicle (Veh) or 5-HTP at different doses 1, 12.5, 25, 50, and 100 mg/kg, pretreated with a fixed dose of CB (75 mg/kg). The horizontal dotted line (at 0 value) represents no weight change relative to BL. *Lower panel:* Dose-response curve of 5-HTP/CB, from the same data shown in the upper *panel*. Each dot indicates the average weight loss achieved across seven consecutive days of treatment. The average weight loss was normalized to the dose that induced the maximum weight loss (gray dashed line 100%). Arrows show the computed effective-dose in mg/kg (ED). **(B)** The change in body weight after Veh or Phen at the following doses of 1, 3, 10, 30, and 45 mg/kg. Symbols represent the mean ± sem. *Lower panel:* Dose-response curve of Phen alone. Each dot indicates the weight loss induced by the different doses of Phen. Values below 0 indicate doses where weight gain was observed (e.g., see #).

To characterize, in rats, the efficacy of 5-HTP/CB on weight loss, we first performed a dose-response curve. **Figure 1A** (*upper panel*) depicts the change in body weight (g) following seven consecutive days after systemic administration of vehicle (open circle) or 5-HTP at doses of 1, 12.5, 25, 50, or 100 mg/kg. As expected, rats treated with the vehicle steadily gained body weight. In contrast, the weight loss induced by 5-HTP/CB followed a nearly dose-dependent response [RM ANOVA; main effect of doses: *F*_(5,21)_ = 30.3, *p* < 0.0001, days 1-7: *F*_(5,6)_ = 10.2, *p* < 0.0001 and doses x days interaction: *F*_(21,126)_ = 5.3, *p* < 0.0001]. Except for 12.5 and 25 mg/kg that changed body weight with the same magnitude (p = 0.85), 5-HTP100/CB induced the maximum weight loss when compared relative with vehicle (see dashed line **Figure 1A** *lower panel*). **Figure 1A** (*lower panel*) shows the dose-response curve for 5-HTP/CB. From these plots, we then obtained the ED_45_ of 5-HTP/CB by using the software of Gadagkar and Call (2015), which corresponds to 31 mg/kg. Thus, in all subsequent experiments, we decided to use the ED_45_ dose, based on a previous study that used a similar 5-HTP dose to evaluate its effects in combination with d-amphetamine (Baumann et al., 2011).

#### Dose-Response Relationship of Phen and Weight-Loss

We performed the same experiment but only using different doses of Phen. As expected, body weight gain was observed in the control group (open circles **Figure 1B**, upper panel), whereas Phen, at 1 mg/kg, induced weight gain (1 mg/kg; see # symbol *lower panel*), 3 mg/kg was not different relative to vehicle, but 10 mg/kg maintained the same body weight relative to the initial weight in BL and it was significantly different relative to vehicle (*p* = 0.047). In contrast, higher doses of Phen 30 and 45 mg/kg induced a significant weight loss when compared against both the vehicle and the initial body weight at BL [RM ANOVA; main effect of doses: *F*_(5,24)_ = 31.5, *p* < 0.0001]. For Phen, the dose of 30 mg/kg (rather than 45 mg/kg) induced the maximum body weight loss (see dashed line **Figure 1B**, *lower panel*), suggesting that 45 mg/kg accelerated the development of pharmacological tolerance (see days 5–7, *upper panel*). The *lower panel* of **Figure 1B** depicts the ED_41_ (10 mg/kg), ED_50_ (15 mg/kg), and ED_56_ (20 mg/kg) for Phen alone.

To evaluate the effects on weight loss, food intake, and locomotion of the triple-drug combination, we decided to combine the most commonly used concentrations reported in literature of Phen (Baumann et al., 2000; Roth et al., 2008; Kalyanasundar et al., 2015) that correspond to ED_41, 50, or 56_ (Phen10, Phen15, or Phen20, respectively) with a fixed ED_45_ dose of 5-HTP/CB (31 and 75 mg/kg, respectively).

A visual inspection of the overall pattern of weight loss induced by 5-HTP/CB or Phen alone, revealed a prominent weight loss initially (**Figure 1**; on days 1–3), whereas, on subsequent days (days 4–7), the weight loss either plateaued or, in some cases, a weight gain was later observed, suggesting the development of pharmacological tolerance. For this reason, in further analysis, we explored data as a function of these two blocks.

#### The Phen + 5-HTP/CB Combination Leads to Greater Weight-Loss and Lower Food Intake Than Either Treatment Alone

**Figure 2:**
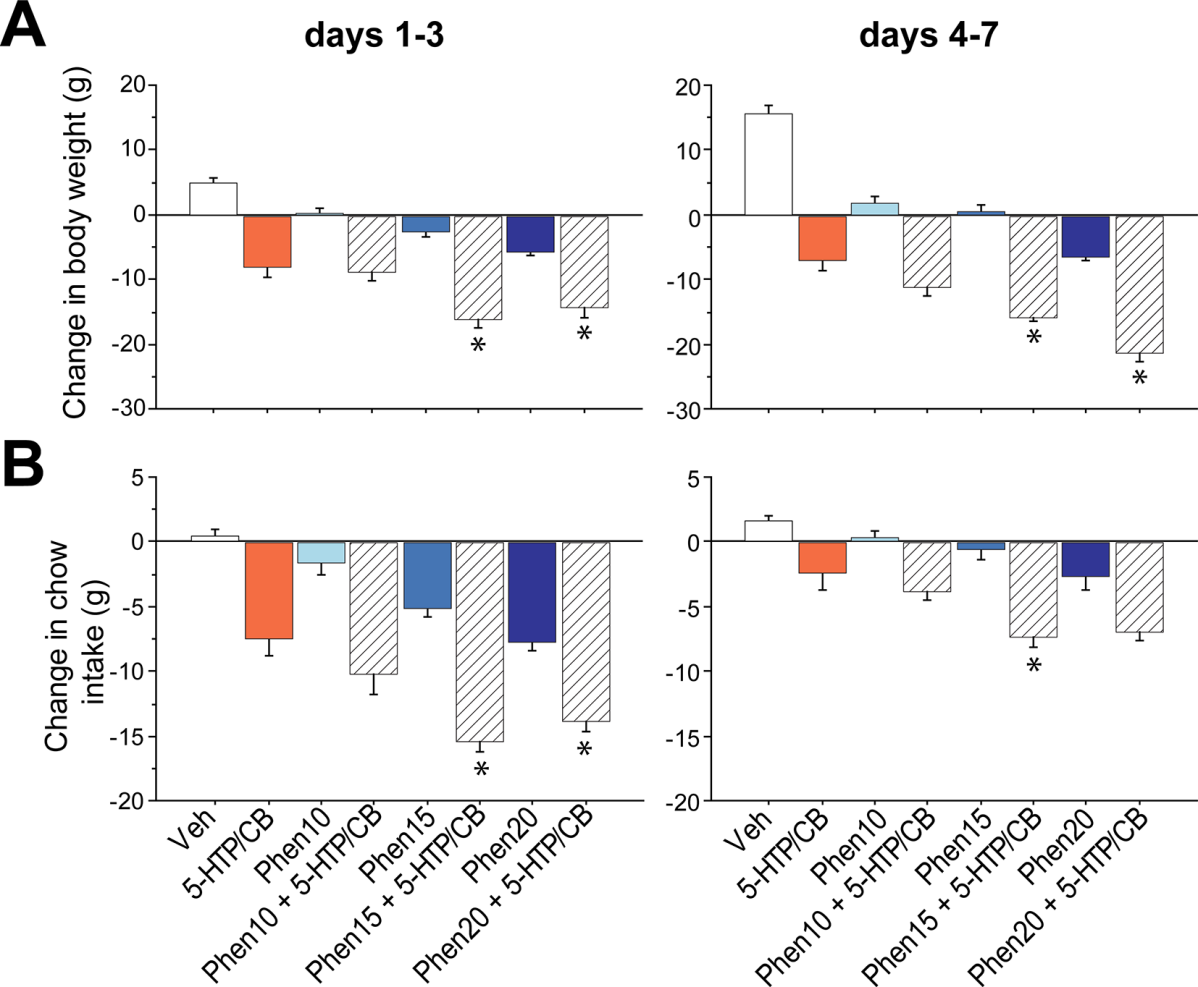
The phentermine (Phen) + 5-hydroxytryptophan (5-HTP)/carbidopa (CB) combination induces greater weight loss and further suppresses food intake than each drug alone. **(A)** *Left panel:* The change in body weight (g) achieved by a combination of Phen + 5-HTP/CB and each treatment alone after the first three days of injection (days 1–3). Control group (Veh), 5-HTP, after a fixed dose of CB pretreatment (ED_45_ 5-HTP = 31 mg/kg and CB = 75 mg/kg), Phen (10, 15, and 20 mg/kg, referred as Phen10, Phen15, and Phen20, respectively) alone and 5-HTP/CB in combination with three different doses of Phen (Phen10 + 5-HTP/CB, Phen15 + 5-HTP/CB, and Phen20 + 5-HTP/CB). *Right panel:* Similar to the left panel but for the second block (days 4–7). **(B)** The change in food intake (g) for the same subjects shown in A for days 1–3 (*left panel*) and on days 4–7 block (*right panel).* Data are mean ± sem. * Indicates values significantly different (*p* 0.05) from both its corresponding Phen dose used in the combination and the 5-HTP/CB group.

Next, we examined whether the triple drug combination induces greater weight loss than either of them alone. **Figure 2A** indicates the change in body weight (g) on days 1–3 block (*left panel*). After injections of the vehicle (control), rats exhibited a weight gain (white bar). In contrast, the 5-HTP/CB group lost weight -8.1 ± 1.5 g (orange). Likewise, the weight loss produced by Phen was dose-dependent (blue bars; 0.1 ± 0.7 g, -3.7 ± 0.8, and -5.7 ± 0.5 g for Phen10, 15, and 20, respectively). However, rats treated with the triple combination lost significantly more weight -8.9 ± 1.4 g, -16.2 ± 1.1, and -14.4 ± 1.4 for Phen10 + 5-HTP/CB, Phen15 + 5-HTP/CB, and Phen20 + 5-HTP/CB, respectively [RM ANOVA main effect treatments *F*_(7,55)_ = 29.3, *p* < 0.0001]. A *post hoc* analysis revealed that Phen15 + 5-HTP/CB and Phen20 + 5-HTP/CB induced greater weight loss than both 5-HTP/CB and Phen alone (All *p* *_s_* < 0.05; see * **Figure 2A**). Thus, our results demonstrate that the triple combination leads to greater weight loss than did either treatment alone.

In the late block days 4–7 (**Figure 2A**, *right panel*), we observed what looks like pharmacological tolerance. This can be seen more clearly in Phen10 and Phen15 as both groups started exhibiting some weight gain. Nevertheless, they still maintained a lower body weight relative to vehicle-treated rats (white bar). In contrast, Phen10 + 5-HTP/CB, Phen15 + 5-HTP/CB, and Phen20 + 5-HTP/CB continued losing weight [RM ANOVA; *F*_(7,55)_ = 34.2, *p* < 0.0001]. Moreover, Phen15 + 5-HTP/CB and Phen20 + 5-HTP/CB groups lost significantly more weight than those treated with each drug alone (All *p*_s_ < 0.05).

Appetite suppressants, as its name indicates, induce weight loss, in part, by suppressing food intake (Adan et al., 2008; Valsamakis et al., 2017; Srivastava and Apovian 2018). Thus, as expected, the weight loss was accompanied by suppression of food intake. On days 1–3 (**Figure 2B**, *left panel*), as expected, we found that the administration of the vehicle did not change food intake (i.e., values remained near to zero; 0.5 ± 0.4 g), while 5-HTP/CB suppressed food intake (-7.4 ± 1.3 g). Likewise, Phen reduced food intake in a dose-dependent manner (blue bars: Phen10; -1.6 ± 0.8, Phen15; -5.1 ± 0.6, and Phen20 -7.7 ± 0.6 g, respectively). Moreover, the triple drug combination achieved the maximum suppression of food intake (-10.1 ± 1.6 g, -15.4 ± 8, and -13.8 ± 0.7, for Phen10 + 5-HTP/CB, Phen15 + 5-HTP/CB, and Phen20 + 5-HTP/CB, respectively) [RM ANOVA; treatment: *F*_(7,55)_ = 30.8, *p* < 0.0001]. Both Phen15 + 5-HTP/CB and Phen20 + 5-HTP/CB reduced food intake more than either treatment alone (All *p* *_s_* < 0.05; see * **Figure 2B** hatched bars). In the late block, on days 4–7 (**Figure 2B**, *right panel*), all groups increased food intake relative to days 1–3, suggesting the development of pharmacological tolerance. Nevertheless, they still exhibited reduced food intake in comparison with vehicle treated rats [RM ANOVA; *F*_(7,55)_ = 8.7, *p* < 0.0001]. Only Phen15 + 5-HTP/CB suppressed food intake more than both Phen15 (*p* = 0.0009) and 5-HTP/CB (*p = 0.03*) groups. These results demonstrate that the triple drug combination suppressed food intake more and for a few more days than either treatment alone.

#### The Phen + 5-HTP/CB Combination Partially Reversed Psychomotor Side-Effects of Phen Alone

**Figure 3:**
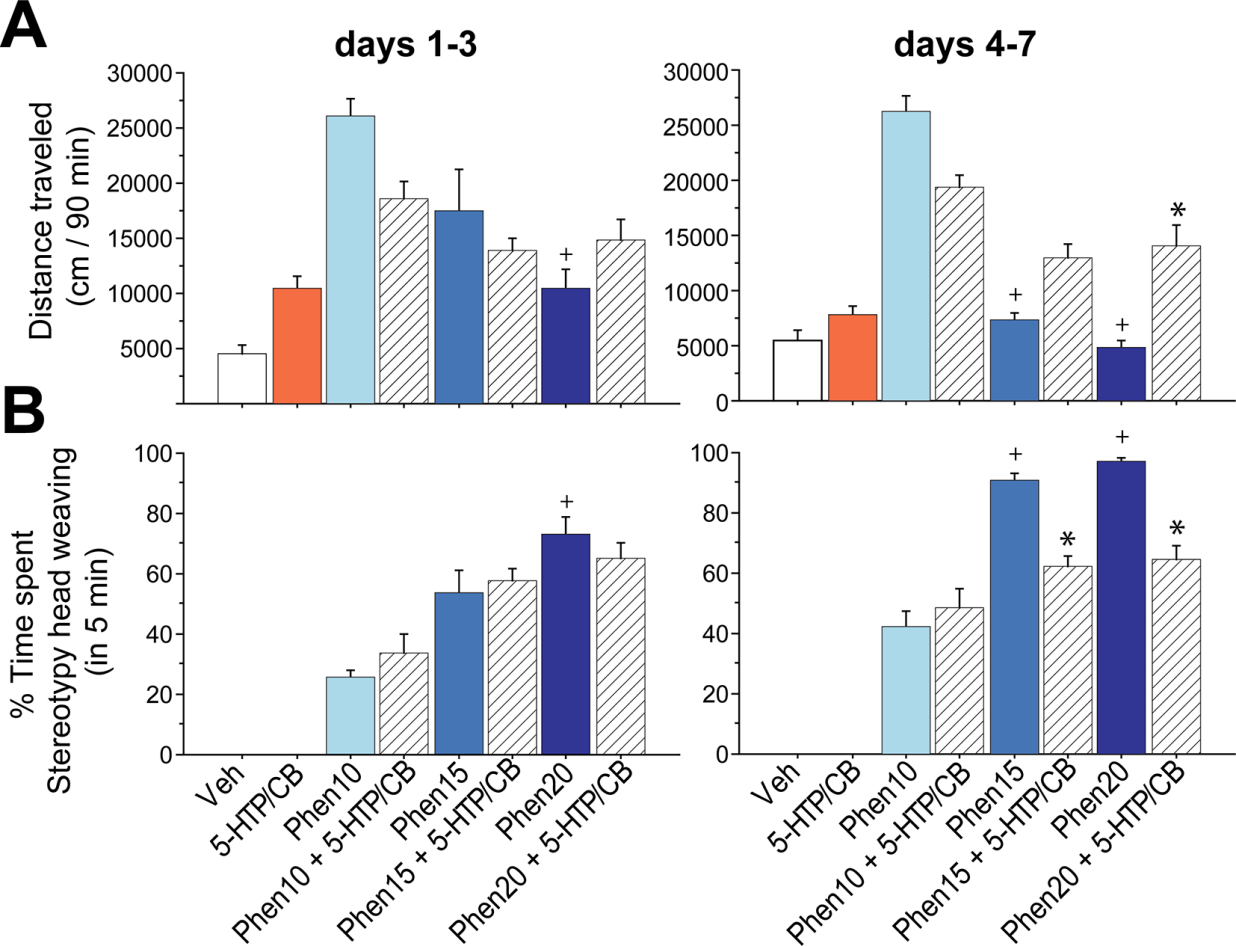
The triple drug combination partially reverses psychomotor side effects (locomotion and stereotypy) of Phen. **(A)** *Left panel:* the total distance traveled (cm/90 min), forward locomotion, across the first three days (days 1–3), and last four days (days 4–7, *right panel*) after injection of vehicle or treatment. Same conventions as in **Figure 2**. **(B)** *Left panel*: cumulative duration, in percentage, of stereotypy during 5 min measured after 1 h from onset of injection for days 1–3 and days 4–7 blocks (*right panel*). Data are mean ± sem. ^+^ *p* 0.05 relative to Phen10, *p 0.05 indicates values significantly different from its corresponding Phen dose used in the combination.

Since it is well known that in rodents appetite suppressants with dopaminergic action stimulate locomotion and stereotypy (Santamaría and Arias, 2010; Ferragud et al., 2014; Kalyanasundar et al., 2015), we asked whether 5-HTP/CB could antagonize the stimulant properties of Phen. To this end, we analyzed the total distance traveled, on days 1–3, and plotted in **Figure 3A** (*left panel*). Both the vehicle and 5-HTP/CB groups exhibited low levels of locomotion; this is because vehicle-treated rats spent most of the time sleeping, while rats treated with 5-HTP/CB were in a quiet awake state (**Supplementary Figure 2**). In contrast, Phen at 10 mg/kg induced a robust hyper-locomotion, but higher doses, Phen15 and Phen20, resulted in hypo-locomotion, indicating an impairment in movement [RM ANOVA, treatment: *F*_(7,51)_ = 5.7, *p* < 0.0001]. A *post hoc* test further confirmed that Phen20 showed less locomotion than Phen10 (*p* = 0.007). For the triple combination, and on days 1–3, we found that Phen10 + 5-HTP/CB group had a lower distance traveled than Phen10, but differences did not achieve significance (*p* = 0.6). A similar trend was observed for Phen15 + 5-HTP/CB relative to Phen15. In contrast, Phen20 + 5-HTP/CB exhibited more locomotion than Phen20 alone, but no significant differences were found (*p* = 0.7).

On days 4–7 block (**Figure 3A**, *right panel*), groups treated with Phen15 and 20 further reduced their locomotion, in particular, the immobility was stronger for Phen15 and Phen20 relatives to Phen10 (All *p* *_s_* < 0.0001). In contrast, Phen20 + 5-HTP/CB displayed more locomotion than Phen20 alone, suggesting that for higher doses (e.g., Phen > 15 mg/kg that elicited hypo-locomotion), the triple combination partially recovered rat’s mobility.

Regarding stereotypy, either vehicle or 5-HTP/CB groups displayed no stereotypy at all (**Figure 3B**), confirming the idea that synaptic release of 5-HT alone is insufficient to stimulate forward locomotion or stereotypic behaviors (Baumann et al., 2011). In contrast, in the first block, Phen evoked the opposite effect on stereotypy than in locomotion. Phen decreased distance traveled (**Figure 3A**) but increased the stereotypy at higher doses (**Figure 3B**, *left panel*; blue bars). In the second block, the stereotypy further increased, especially for Phen15 and Phen20 relative to Phen10 treated group (days 4–7; **Figure 3B**, *right panel*). Altogether, our data indicate that the hypo-locomotion evoked by higher doses of Phen was due to a heightened in repetitive movements (i.e., head weavings).

We observed that during the first three days of treatment, the triple drug combination, at doses tested, did not attenuate stereotypy (**Figure 3B** see dashed bars). In contrast, on days 4–7 (*right panel*), Phen15 + 5-HTP/CB and Phen20 + 5-HTP/CB exhibited a significant reduction in stereotypy relative to its corresponding Phen group (Phen15, *p* = 0.008; Phen20, *p* = 0.002), substantiating that, as the treatment progress, 5-HTP/CB partially reverses psychomotor side-effects of Phen.

### Electrophysiology

#### Phen Inhibits NAcSh Responses

**Figure 4:**
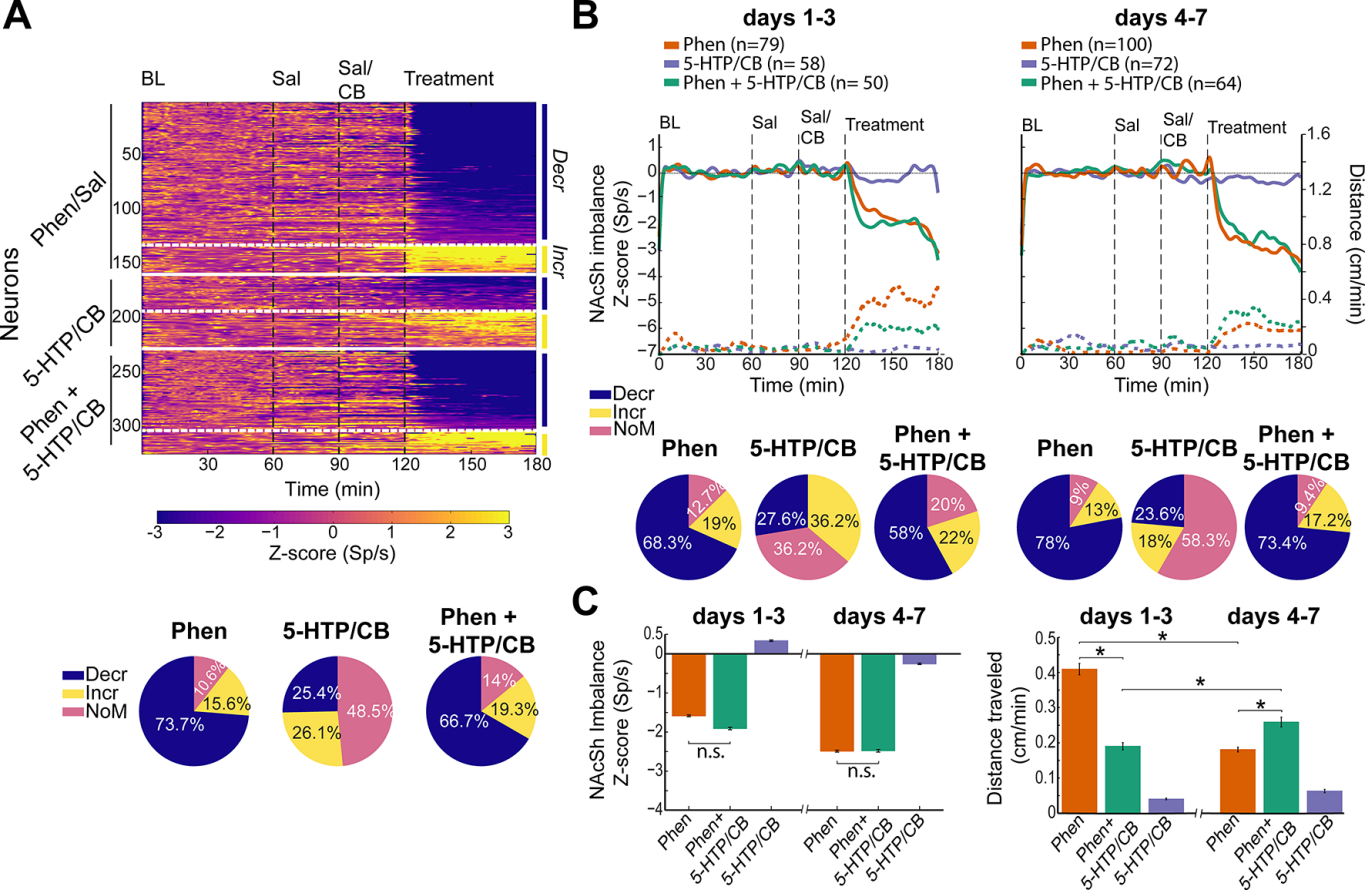
Phentermine (Phen) induces an inhibitory imbalance in population nucleus accumbens shell (NAcSh) activity, and the addition of 5-hydroxytryptophan (5-HTP)/carbidopa (CB) did not reverse the imbalance. **(A)** *Upper panel:* population activity of 325 (out of 423) neurons recorded during 180 min spanning four different epochs including the baseline (BL), Saline (Sal), CB (in the case of 5-HTP), and treatments (Phen, or 5-HTP, or Phen + 5-HTP/CB) indicated by the vertical dotted lines. The activity of each neuron was normalized to z-score relative to BL epoch and plotted in a color-coded peri-stimulus time histogram (PSTH). The horizontal solid white lines separated the neuronal modulations evoked by different drug treatments, whereas the dotted white line separated the NAcSh neuronal activity that decreased (Decr) or increased (Incr) after treatment. *Lower panel*. Percentage of neurons that decreased or increased firing rate significantly, and non-modulated (NoM) neurons after treatment. **(B)** The population firing rate (normalized to z-score) in each epoch as a function of days 1-3 block (*left panel*) and on days 4–7 block (*right panel*). Note that negative z-scores indicate an inhibitory imbalance (right x-axis). Dashed lines depict the locomotion induced by each treatment (see units in right y-axis). *Lower panel*. Pie chart of modulation of each treatment for the two blocks. **(C)** *Left panel*, overall average activity 50 min after onset of drug injection (from 130 to 180 min in panel B), as a function of blocks. *The right panel* depicts the distance traveled in the same time interval for each treatment. * Significantly different with an alpha 0.05. Percentages may not total 100% because of rounding.

To characterize how these antiobesity drugs modulate NAcSh responses, we recorded extracellular single-unit activity, while rats received, *via* an intraperitoneal catheter, infusions of the abovementioned appetite suppressants. A total of 423 neurons were recorded in NAcSh while rats were infused with either Phen (15 mg/kg), or 5-HTP/CB (31/75 mg/kg), or Phen + 5-HTP/CB. **Supplementary Figure 3** shows the approximate location of electrode tracks targeting the NAcSh. **Figure 4A** displays the normalized NAcSh neuron activity in color-coded PSTH, where yellow indicates activity above BL, and inhibitions are in blue. Neuronal activity is depicted as a function of the following four epochs: 1) BL, 2) Saline (Sal), 3) Sal/CB, or 4) Drug treatment, which is Phen, or 5-HTP, or Phen + 5-HTP. Two types of modulatory responses were observed: neurons whose activity decreased (Decr; blue color area) and those whose activity increased relative to BL (Incr; yellow color area). Within a few minutes from the onset of treatment, the majority of NAcSh neurons exhibited a large decrease in spiking (see Decr; blue colors after 120 min), while a smaller population increased their responses after the infusion of Phen (see Incr; yellow). After Phen, 73.7% (132/179) of NAcSh neurons were classified as Decr, while 15.6% (28/179) were Incr (χ2 = 48.6, *p* < 0.0001; Decr vs. Incr): and the other 10.6% were NoM (**Figure 4A**, see pie chart). Overall, Phen modulates 89.3% of NAcSh neurons recorded, with the majority exhibiting a long-lasting inhibition for at least 1 h.

Unlike Phen, 5-HTP/CB modulated nearly the same number of neurons with either decreasing 25.4% (33/130) or increasing responses 26.1% (34/130) (χ2 = 0.01, *p* = 0.9). Thus, this serotonin precursor did not produce any inhibitory imbalance at the population activity level. Nevertheless, more than half (51.5%) of NAcSh neurons were sensitive to 5-HTP/CB.

Similar to Phen, in the case of the triple combination, we also observed a higher proportion of neurons with Decr (66.7%, 76/114) than Incr responses (19.3%, 22/114)(χ2 = 21.4, *p* < 0.0001), revealing that the infusion of Phen + 5-HTP/CB did not alter the Phen evoked inhibitory imbalance in NAcSh responses.

The normalized population activity (relative to BL) of all neurons pooled together across the first three (1–3) or last four (4–7) days can be seen in **Figure 4B** (*upper panel)*. For the three groups, and after infusion of saline or CB, the activity did not significantly change relative to BL epoch. Likewise, infusion of 5-HTP/CB alone did not alter the population’s activity balance, and if any, it can be observed a slight increase in neural activity around 30 min after infusion (at time 150 min, purple line; *left panel*). In contrast, the infusion of Phen (orange) or the triple drug combination (green; Phen + 5-HTP/CB at time 120 min) within minutes evoked a large inhibitory imbalance in population NAcSh activity. The inhibitory imbalance induced by both Phen and the triple combination was of similar magnitude (Kruskal-Wallis test; χ2_(2)_ = 25, *p* = n.s.), suggesting that the triple combination did not further enhance, or reduce, the imbalance (**Figure 4C**; *left panel, days 1-3*). Thus, our results suggest that the greater weight loss induced by the triple drug combination was not explained by the net inhibitory imbalance in the NAcSh population activity.

A similar modulatory pattern was observed on days 4–7. No significant differences were observed between Phen and Phen + 5-HTP/CB groups (Kruskal-Wallis test; χ2_(2)_ = 43.8, *p* = n.s.; **Figures 4B, C**), confirming once more that the combination did not alter the Phen-induced inhibitory imbalance of NAcSh responses.

We note that on the late block- relative to the first one- the infusion of 5-HTP/CB now induced a slight inhibition in NAcSh responses (purple, **Figures 4B, C**), although it was always of less magnitude than that of Phen. The percentage of neurons modulated in each block can be seen in the pie charts below in **Figure 4B**. We note that the higher the inhibitory imbalance evoked by 5-HTP/CB, on days 4–7, would be rationalized, in part, by a trend towards significance in the drop of neurons with increasing responses from 36.2% (21/58) to 18% (13/72) from first to second block (χ2_(2)_ = 3.175, *p* = 0.07).

Since Phen stimulates locomotor activity (**Figure 3A**), we then investigated whether NAcSh responses would correlate with locomotion. **Figure 4B** (dashed lines) plots the forward locomotion across the session. It can be appreciated that infusion of Phen either alone or in combination induced both an increase in locomotor activity and, in parallel, the inhibitory imbalance in NAcSh responses (**Figure 4B** compare dashed lines (i.e., locomotion) vs. solid lines (i.e., population NAcSh activity)). In fact, we found a negative correlation between locomotion and the Phen-induced NAcSh inhibitory imbalance (Pearson correlation on days 1-3: r = -0.91, *p < 0.001;* on days 4–7: r = -0.92, *p <0.001*). A similar negative correlation was found for the Phen-5-HTP/CB-induced activity imbalance (days 1–3: r = -0.85, *p < 0.001*; on days 4–7: r = -0.76, *p < 0.001*). In contrast, 5-HTP/CB neither stimulated locomotion nor induced any inhibitory imbalance. In that sense, both the locomotion and NAcSh’s inhibitory imbalance always covaried. That said, it could also be seen that its magnitude did not change in the same proportion as the magnitude of forward locomotor activity. For example, on days 1–3, Phen stimulated the strongest locomotor activity, but on days 4–7 locomotion was lower (orange bars, **Figure 4C**, *right panel*) (Mann Whitney test; U = 38076, *p* < 0.0001), while Phen + 5-HTP/CB showed the opposite pattern that is low locomotion on days 1–3 but more on days 4–7 (see green bars)(Mann Whitney test; U = 147306, *p* < 0.0001). This is despite that Phen and Phen + 5-HTP/CB both evoked a similar NAcSh’s inhibitory imbalance in both blocks (compare early vs. late) (**Figure 4C**, *left panel*). Thus, our data suggest that NAcSh’s inhibitory imbalance gates (or triggers) locomotor activity, rather than directly determines its magnitude.

**Figure 5:**
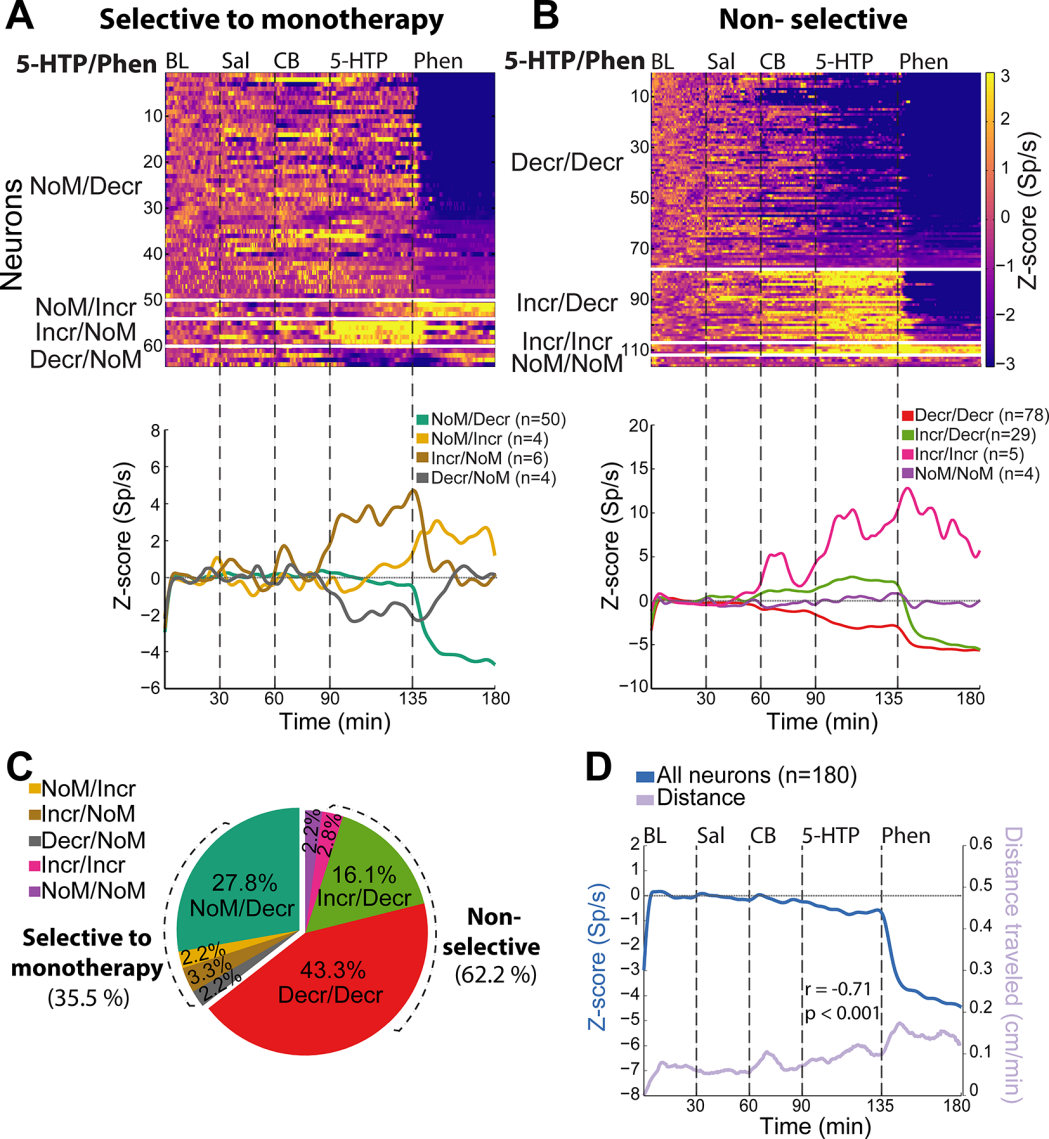
The majority of nucleus accumbens shell (NAcSh) neurons modulated by 5-hydroxytryptophan (5-HTP) are further suppress by phentermine (Phen). **(A)** *Upper panel:* A color-coded peri-stimulus time histogram (PSTH) plotted NAcSh neurons with selective responses to either 5-HTP or Phen. The vertical dotted lines indicated each epoch (baseline, BL; saline, Sal; carbidopa, CB; 5-HTP, and Phen. *Lower panel:* Population PSTH of all neurons with selective responses to one drug (NoM: Nonmodulated response, Decr: Decrease response, Incr: Increase response). **(B)** Ensembles of neurons with non-selective responses, same conventions as in panel **(A)**. **(C)** Percentage of neurons for each modulatory pattern displayed in panels **(A** and **B)**. **(D)** Normalized population PSTH of all neurons recorded in NAcSh (blue line; left y-axis) and the total distance traveled across the session (purple line; right y-axis). The Pearson correlation coefficient between locomotion and NAcSh population activity (from 30 to 180 min) is also shown.

Finally, we tested whether 5-HTP/CB and Phen targets either the same or different NAcSh ensembles. To this end, we implanted a new group of rats with an electrode array in the NAcSh and administered all these three drugs in the same session but at different time epochs. For this experiment, we record a total of 180 NAcSh neurons. **Figure 5A** displays a color-coded PSTH of all neurons, exhibiting a selective response to one drug and its sign of modulation. We classified responses either as NoM, meaning not-modulated, Decr, or Incr as a function of 5-HTP or Phen. For example, the first ensemble, in **Figure 5A**, described a subpopulation of neurons with NoM/Decr (5-HTP/Phen) modulatory pattern, meaning that it did not respond to 5-HTP, but its activity decreased after Phen. In this regard, the Phen-selective population was the largest among neurons with responses selective to one drug (NoM/Decr; 27.8% neurons). In contrast, only a few neurons were selective to 5-HTP/CB (Incr/NoM; 3.3% and Decr/NoM; 2.2%).

Our results showed that it was far more common to record nonselective than selective responses (i.e., responding to both drugs 62.2%, 112/180 vs. those responding selectively to one drug 35.5%, 64/180; **Figures 5A, B**), suggesting that both drugs mainly recruited the same NAcSh neurons. The largest group belongs to the Decr/Decr modulatory pattern (43.3%, 78/180). In comparison with the experiment depicted in **Figure 4** (5-HTP/CB Decr 25.4%), we noted that the increase in Decr neurons induced by 5-HTP/CB (**Figure 5**, 43.3%) could be explained, in part, by a potential carry-over effect of Phen across consecutive days. Furthermore, we observed that even neurons with increasing responses to 5-HTP/CB were further inhibited by Phen (see Incr/Decr, 29/180; 16.1%). **Figure 5C** depicts the percentage of neurons belonging to each population. Altogether, our results rationalized how the combination Phen + 5-HTP/CB evokes a change in firing rates (increase/decrease) toward an inhibitory imbalance in NAcSh responses that seems to gate locomotion (**Figure 5D**).

## Discussion

Obesity is a rapidly growing public health problem affecting an increasing number of people worldwide. Previously, we characterized Phen and two other dopaminergic appetite suppressants (Kalyanasundar et al., 2015). Here, we have extended these studies to 5-HTP a serotoninergic compound. In this regard, Hendricks et al., 2009 reported that obesity specialists frequently prescribed, in humans, a triple drug combination (Phen, 5-HTP, and CB) for the treatment of obesity. However, there is no preclinical study confirming its efficacy neither its evoked effects in the brain. Our findings in rats support the potential use of Phen + 5-HTP/CB as an antiobesity drug combination. Relative to the individual treatments of Phen and 5-HTP/CB, the triple drug combination leads to greater weight loss and a robust food intake suppression while partially reducing adverse psychomotor side effects. Electrophysiological recordings from the NAcSh further revealed that drugs mostly recruited the same neuronal ensembles. However, the Phen-induced inhibitory NAcSh responses were more prominent than those induced by 5-HTP/CB. Consequently, both Phen alone and in combination with 5-HTP/CB always evoked a similar inhibitory imbalance in NAcSh population responses, resembling the effects of Phen alone. Thus, our data suggest that other brain regions, outside the NAcSh, could be responsible for the greater weight loss reported herein. Furthermore, this is the first demonstration that Phen and 5-HTP/CB recruits largely overlapping neuronal populations, and thus, it paves the way to understand how appetite suppressants that act *via* DA or 5-HT neuromodulate NAcSh ensemble activity.

### The Triple Drug Combination as a Potential Treatment for Overweight and Obesity

Dopamine and serotonin are two relevant neurotransmitters involved in food intake and body weight homeostasis (Meguid et al., 2000; van Galen et al., 2018). Therefore, from a therapeutic standpoint, the interaction DA/5-HT would be useful to understand. Our study revealed that the triple drug combination of Phen and 5-HTP/CB leads to a greater weight loss and stronger food suppression than each drug alone (**Figure 2**), supporting previous studies suggesting that DA releasing agents and 5-HT precursor drugs induce greater pharmacological effects than each drug alone (Halladay et al., 2006; Baumann et al., 2011).

Although the precise molecular mechanism responsible for DA/5-HT interaction is currently unknown, the 5-HT_2C_ receptor is a likely candidate. In this regard, in humans and rats, the combination Phen + Lorcaserin (a selective 5-HT_2C_ agonist) also induced greater weight loss and robust food suppression than either treatment alone (Grottick et al., 2015; Smith et al., 2017). Another potential candidate could be the 5-HT_1B_ receptor (Lucas et al., 1998). Our results then raise the possibility that the combination of 5-HTP/CB with other appetite suppressants of the same class as Phen (e.g., diethylpropion and bupropion, to name a few) (Kalyanasundar et al., 2015) would also exhibit greater pharmacological effects upon weight loss and food intake. However, this remains to be proven experimentally.

We highlight that our study is of preclinical nature. However, we note that 5-HTP is a clinically-effective serotonin precursor, available for human use as an over-the-counter supplement, and it has not been associated with dangerous side effects, including the induction of the serotonin syndrome (Gijsman et al., 2002; Turner et al., 2006). Also, since our combination used CB to block the conversion of 5-HTP into 5-HT, a reduction in peripheral side effects, this result was expected. Nevertheless, we cannot rule out this potential side effect after chronically challenging the serotonergic system. Moreover, since 5-HTP is not considered a 5-HT releaser, it is expected to have a different toxicity profile than fenfluramine, which was withdrawn from the market for producing cardiac valvopathy (McMurray et al., 1986; Brenot et al., 1993). CB is commonly used to treat Parkinson’s symptoms with L-DOPA/CB combination (Mooney et al., 2007; Schneider et al., 2018). Likewise, Phen is currently approved for the short-term treatment of overweight and obesity (Valsamakis et al., 2017). However, appropriate safety studies are needed to translate this combination into the clinic.

### 5-HTP/CB Can Reduce Motor Stimulant Properties of Phen

Motor stereotypies are unnecessary repetitive behaviors that can be induced with repeated or high doses of psychomotor stimulants (Fibiger et al., 1973; Antoniou et al., 1998; Santamaría and Arias 2010; Ferragud et al., 2014). The role of DA in the expression of stereotypy is well established (Chartoff et al., 2001; Engeln et al., 2019). We suggested that motor stereotypies can be used as an indirect measurement of the ability of an appetite suppressant to challenge the dopaminergic system (Kalyanasundar et al., 2015). The stimulant properties of Phen were complex since locomotion (distance traveled) decreased, while stereotypy (head weavings) increased as the dose of Phen increased from 10 to 15 or 20 mg/kg (**Figure 3**). The specific receptors responsible for these effects are unknown, but DA receptors are likely candidates. This is because blockade of D1 or D2-like receptors either *via* systemic intragastric or direct infusion in the NAcSh attenuated the stimulant properties of diethylpropion, an appetite suppressant itself as well as an analog of Phen (Kalyanasundar et al., 2015), Therefore, we propose that similar to diethylpropion, DA receptors located in NAcSh could also underlie the stimulant properties of Phen.

Furthermore, we found the combination of Phen + 5-HTP/CB partially reversed the stimulant properties of Phen. Briefly, for Phen10, the combination reduced motor activity whereas, for Phen15 and Phen20, rat’s locomotion was partially recovered, and the stereotypy was attenuated (**Figure 3**). Altogether, our results demonstrate that 5-HTP, after CB pretreatment, can partially reverse the adverse psychomotor properties of Phen, especially in the late phase of administration (days 4–7; **Figures 3A, B**). Our results are in agreement with previous studies, indicating that 5-HTP can reduce the motor stimulant properties of d-amphetamine, without altering the DA-releasing properties of the drug (Baumann et al., 2011).

The mechanisms and the specific 5-HT receptor subtype, which might be involved with the antagonism of Phen-induced psychomotor effects are not known, but again 5-HT_2C_ receptors are likely candidates. It is well known that 5-HT_2C_ receptor agonist reduces motor activity produced by stimulant drugs (Di Matteo et al., 2001; Baumann et al., 2011). Further studies should reveal the role of these receptors in the ability of 5-HTP to antagonize stimulant properties of dopaminergic releasing agents.

### How Phen Inhibits Spiking Activity in the NAcSh?

Our electrophysiological recordings yield insights into the firing rate homeostasis in the NAcSh responses. Previous studies, in mice, have shown that even after a strong optogenetic stimulation of glutamatergic afferent inputs, activate and inactivate neurons in a manner that maintains firing rate balance (homeostasis) at the NAcSh population level (Prado et al., 2016). In contrast, here, we found that Phen induced a large inhibitory imbalance in NAcSh population activity (also see Kalyanasundar et al., 2015). We suggest that this phenomenon could be mediated by the ability of DA to presynaptically inhibit both excitatory (glutamate) and inhibitory (GABA) release in the NAc (Pennartz et al., 1992; Nicola and Malenka 1997). This presynaptic inhibition is important for understanding how DA affects NAc neuron activity because MSNs do not fire in the absence of excitatory afferent activity (Higashi et al., 1989). Nevertheless, this idea remains to be experimentally proven. This could be tested by using fiber photometry and membrane-bound proteins that emit green fluorescence in the presence of glutamate (sniffer protein iGluSnFR) (Marvin et al., 2013), DA (D-Light) (Patriarchi et al., 2018), or GABA (iGABASnFR) (Bras, 2019).

### Lack of Synergism on NAcSh’s Neuronal Modulations Induced by Phen and 5-HTP/CB

Our findings are in agreement with three previous studies, using *in vivo* microdialysis in rats, implicating the lack of synergism in DA release between DA and 5-HT drugs. In a series of elegant studies, Rothman’s group found no evidence of synergy (or at best a marginal effect) in NAc DA release when the combinations Phen + Fenfluramine (a 5-HT releaser), Phen + 5-HTP (pretreated with benserazide), and d-amphetamine + 5-HTP (Baumann et al., 2000; Halladay et al., 2006; Baumann et al., 2011) were given as a mixture. Altogether, this data supports the notion that 5-HT agents did not alter the ability of Phen to release DA in the NAc. Regarding our electrophysiological recordings, our study showed no evidence of synergism between Phen and 5-HTP/CB but in the electrophysiological NAcSh responses evoked by Phen. Moreover, Halladay et al. (2006) demonstrated that Phen and 5-HTP evoked simultaneous elevations in extracellular DA and 5-HT levels in the NAc that mirrored the effects of each drug alone. Nevertheless, we acknowledge the possibility that DA and 5-HT could still interact in the NAcSh. That is, we cannot rule out a DA/5-HT interaction since we did not measure these neurotransmitters and because there is evidence indicating that 5-HT_2_ receptor agonists enhance 3,4-methylenedioxymethamphetamine (MDMA)- DA release (Huang and Nichols 1993; Gudelsky et al., 1994). Moreover, amphetamine-induced DA release in the NAc is reduced by a selective 5-HT_2A_ antagonist (Porras et al., 2002). In contrast, 5-HT_2C_ receptors modulate the mesolimbic DA system but in the opposite direction than 5-HT_2A_ receptors (Di Matteo et al., 1999; Giovanni et al., 2000; Di Matteo et al., 2001). Together, these studies raise the possibility that DA and 5-HT could interact. Further studies are needed to evaluate this possibility.

### Other Brain Regions Outside the NAcSh Would Be Responsible for the Greater Pharmacological Effects on Food Intake Suppression and Weight Loss

As noted, previous studies, using microdialysis, have failed to find synergism between DA and 5-HT release (Halladay et al., 2000; Halladay et al., 2006). Likewise, we found no evidence of synergism in the net inhibitory imbalance in NAcSh responses evoked by the triple combination (**Figure 4**), suggesting that brain circuits outside the NAcSh could be responsible for this pharmacological interaction. At this point, it is only possible to speculate, but recent advances in optogenetics have confirmed the participation of two main groups of orexigenic neurons the AgRP in the arcuate nucleus (the homeostatic feeding system) and the GABAergic neurons in the lateral hypothalamus area (the hedonic feeding system). That is, optogenetic stimulation of both these neurons promotes voracious feeding (Stuber and Wise, 2016; Sternson and Eiselt, 2017). Likewise, anorectic POMC neurons in the arcuate nucleus are also interesting candidates since POMC neurons express 5-HT_2C_ receptors (Xu et al., 2008; Garfield and Heisler, 2009), and they mediate the anorectic effects induced by 5-HT agents, including lorcaserin (Thomsen et al., 2008; Smith et al., 2010; Fidler et al., 2011). Thus, future studies should uncover the participation of these neuronal populations and associated brain circuits, in the mechanism of action of appetite suppressants and its combinations.

#### Phen and 5-HTP/CB Recruit the Same Overlapping NAcSh Ensembles

Although the roles of DA and 5-HT in the ventral striatum are well established for feeding and some neuropsychiatric maladies such as addiction and depression, respectively (Zangen et al., 2001; Wang et al., 2002; Wang et al., 2004; Nautiyal and Hen, 2017), it is unclear how they modulate NAcSh ensemble activity (Klawonn and Malenka, 2019). The appetite suppressant, Phen, is a catecholamine agent that elicits NE and DA release in the brain with lower effects on 5-HT (Balcioglu and Wurtman, 1998; Rothman and Baumann, 2009). In contrast, 5-HTP elevates 5-HT concentration in the brain and to a lesser degree DA release (Turner et al., 2006; Baumann et al., 2011). Thus, each appetite suppressant produces a specific neurochemical profile that most likely determines its desirable pharmacological effects but also its adverse side-effects. Previous studies support the idea that DA releasing appetite suppressants (e.g., Phen) and 5-HT precursors (e.g., 5-HTP) can be coadministered without altering their ability to release DA and increase 5-HT levels, respectively (Halladay et al., 2006; Baumann et al., 2011). Thus, it follows that by mixing different doses, we could tune the DA/5-HT ratio to yield optimal pharmacological effects. Our results are in line with this idea, but at the electrophysiological level, since we found that 5-HTP/CB did not interfere with Phen-induced inhibitory imbalance in NAcSh responses.

Furthermore, and for the first time, we observed that Phen recruits more NAcSh neurons than 5-HTP/CB. Notably, the majority of NAcSh neurons that were sensitive to 5-HTP/CB were further suppressed by Phen. Thus, we rationalized how Phen could induce the strongest modulations either alone or in combination. Importantly, we uncovered that a DA releasing agent and a 5-HT precursor both recruited largely overlapping neuronal ensembles, suggesting that NAcSh neurons received convergent influence of DA and 5-HT afferents.

In summary, we provide preclinical evidence, in rats, supporting the use of a triple drug combination for the treatment of obesity, while partially reducing undesirable psychomotor side-effects of Phen. Our findings contribute to the understanding of how DA and 5-HT acting appetite suppressants modulate NAcSh responses.

## Data Availability Statement

The datasets generated for this study are available on request to the corresponding author.

## Ethics Statement

All procedures were approved by the CINVESTAV institutional animal care and use committee.

## Author Contributions

CP, BK, and RG designed research. CP, MM, and KB performed research. CP analyzed data and made figures. RG wrote the article.

## Funding

This project was supported in part by CONACyT Grants APN 464 and Productos Medix 3247 (to RG).

## Conflict of Interest

Productos Medix SA. de CV. obtained a patent (MX2016011140A) that includes part of the data in this manuscript, and RG is listed as one of the inventors.

The remaining authors declare that the research was conducted in the absence of any commercial or financial relationships that could be construed as a potential conflict of interest.

## Supplementary Material

Supplementary Figure 1 Carbidopa alone prevented body weight gain. The left panel depicts the change in body weight across 7 consecutive days of saline (Sal), control carboxymethyl cellulose (CMC+Sal), or carbidopa CB+Sal (75 mg/kg) injections. Rats treated with CB maintained the same body weight relative to their initial weight in BL. The panel at right indicates the average weight change in g. * *p*< 0.05. Data are mean ± sem.

Supplementary Figure 2 Behavioral states induced by appetite suppressants. Percentage of cumulative time spent in each of the following behavioral states: sleeping, quite awake, and moving for 5 min. Measurements were made around 1 h after the onset of drug administration. Data are mean ± sem.

Supplementary Figure 3 Histological reconstruction of electrodes’ tips in NAcSh. A) Nissl-stained coronal section showing an example of a representative electrode track indicated by the black arrow located in NAcSh. **B)** Summary of NAcSh recording sites across rats. NAcSh, Nucleus Accumbens Shell; cc, Corpus Callosum; aca, Anterior Commissure; CPu, Caudate Putamen (striatum).

## References

[B1] Adan R. A. H. Vanderschuren L. J. la Fleur S. E. (2008). Anti-obesity drugs and neural circuits of feeding. Trends Pharmacol. Sci. 29, 208–217. 10.1016/j.tips.2008.01.008 18353447

[B2] Amer A. Breu J. McDermott J. Wurtman R. J. Maher T. J. (2004). 5-Hydroxy-l-tryptophan suppresses food intake in food-deprived and stressed rats. Pharmacol. Biochem. Behav. 77, 137–143. 10.1016/j.pbb.2003.10.011 14724051

[B3] Antoniou K. Kafetzopoulos E. Papadopoulou-Daifoti Z. Hyphantis T. Marselos M. (1998). d-amphetamine, cocaine and caffeine: a comparative study of acute effects on locomotor activity and behavioural patterns in rats. Neurosci. Biobehav. Rev. 23, 189–196. 10.1016/S0149-7634(98)00020-7 9884112

[B4] Balcioglu A. Wurtman R. J. (1998). Effects of phentermine on striatal dopamine and serotonin release in conscious rats:In vivo microdialysis study. Int. J. Obes. 22, 325–328. 10.1038/sj.ijo.0800589 9578237

[B5] Baumann M. H. Ayestas M. A. Dersch C. M. Brockington A. Rice K. C. Rothman R. B. (2000). Effects of phentermine and fenfluramine on extracellular dopamine and serotonin in rat nucleus accumbens: Therapeutic implications. Synapse 36, 102–113. 10.1002/(SICI)1098-2396(200005)36:2<102::AID-SYN3>3.0.CO;2-# 10767057

[B6] Baumann M. H. Williams Z. Zolkowska D. Rothman R. B. (2011). Serotonin (5-HT) precursor loading with 5-hydroxy-l-tryptophan (5-HTP) reduces locomotor activation produced by (+)-amphetamine in the rat. Drug Alcohol Depend. 114, 147–152. 10.1016/j.drugalcdep.2010.09.015 21071157 PMC3044786

[B7] Beakley B. D. Kaye A. M. Kaye A. D. (2015). Tramadol, pharmacology, side effects, and Serotonin Syndrome: a review. Pain Physician. 18, 395–400. 26218943

[B8] Boudreau E. Chen G. Li X. Buck K. Hitzemann R. Hickman D. (2010). Intraperitoneal catheter placement for pharmacological imaging studies in conscious mice. Lab. Anim. 39, 23–25. 10.1038/laban0110-23 PMC4994513 20023678

[B9] Bras A. L. (2019). A sensor for GABA. Lab. Anim. 48, 263–263. 10.1038/s41684-019-0390-y

[B10] Brenot F. Herve P. Petitpretz P. Parent F. Duroux P. Simonneau G. (1993). Primary pulmonary hypertension and fenfluramine use. Heart 70, 537–541. 10.1136/hrt.70.6.537 PMC1025385 8280518

[B11] Chartoff E. H. Marck B. T. Matsumoto A. M. Dorsa D. M. Palmiter R. D. (2001). Induction of stereotypy in dopamine-deficient mice requires striatal D1 receptor activation. Proc. Natl. Acad. Sci. 98, 10451–10456. 10.1073/pnas.181356498 11517332 PMC56981

[B12] Di Matteo V. De Blasi A. Di Giulio C. Esposito E. (2001). Role of 5-HT2C receptors in the control of central dopamine function. Trends Pharmacol. Sci. 22, 229–232. 10.1016/S0165-6147(00)01688-6 11339973

[B13] Di Matteo V. Di Giovanni G. Di Mascio M. Esposito E. (1999). SB 242 084, a selective serotonin2C receptor antagonist, increases dopaminergic transmission in the mesolimbic system. Neuropharmacology 38, 1195–1205. 10.1016/S0028-3908(99)00047-7 10462132

[B14] Engeln M. Song Y. Chandra R. La A. Evans B. Fox M. E. (2019). Individual differences in stereotypy and neuron subtype translatome with TrkB deletion. bioRxiv, 640987. 10.1101/640987 PMC8480032 32366954

[B15] Ferragud A. Velázquez-Sánchez C. Canales J. J. (2014). Modulation of methamphetamine’s locomotor stimulation and self-administration by JHW 007, an atypical dopamine reuptake blocker. Eur. J. Pharmacol. 731, 73–79. 10.1016/j.ejphar.2014.03.015 24675149

[B16] Fibiger H. C. Fibiger H. P. Zis A. P. (1973). Attenuation of amphetamine-induced motor stimulation and stereotypy by 6-hydroxydopamine in the rat. Br. J. Pharmacol. 47, 683–692. 10.1111/j.1476-5381.1973.tb08194.x 4146741 PMC1776066

[B17] Fidler M. C. Sanchez M. Raether B. Weissman N. J. Smith S. R. Shanahan W. R. (2011). A one-year randomized trial of lorcaserin for weight loss in obese and overweight adults: the BLOSSOM trial. J. Clin. Endocrinol. Metab. 96, 3067–3077. 10.1210/jc.2011-1256 21795446

[B18] Gadagkar S. R. Call G. B. (2015). Computational tools for fitting the Hill equation to dose-response curves. J. Pharmacol. Toxicol. Methods 71, 68–76. 10.1016/j.vascn.2014.08.006 25157754

[B19] Garfield A. S. Heisler L. K. (2009). Pharmacological targeting of the serotonergic system for the treatment of obesity. J. Physiol. 587, 49–60. 10.1113/jphysiol.2008.164152 19029184 PMC2670022

[B20] Gijsman H. J. van Gerven J. M. A. de Kam M. L. Schoemaker R. C. Pieters M. S. M. Weemaes M. (2002). Placebo-controlled comparison of three dose-regimens of 5-hydroxytryptophan challenge test in healthy volunteers. J. Clin. Psychopharmacol. 22, 183–189. 10.1097/00004714-200204000-00012 11910264

[B21] Giovanni G. D. Matteo V. D. Mascio M. D. Esposito E. (2000). Preferential modulation of mesolimbic vs. nigrostriatal dopaminergic function by serotonin2C/2B receptor agonists: a combined *in vivo* electrophysiological and microdialysis study. Synapse 35, 53–61. 10.1002/(SICI)1098-2396(200001)35:1<53::AID-SYN7>3.0.CO;2-2 10579808

[B22] Green E. Jacobson A. Haase L. Murphy C. (2011). Reduced nucleus accumbens and caudate nucleus activation to a pleasant taste is associated with obesity in older adults. Brain Res. 1386, 109–117. 10.1016/j.brainres.2011.02.071 21362414 PMC3086067

[B23] Grottick A. J. Whelan K. Sanabria E. K. Behan D. P. Morgan M. Sage C. (2015). Investigating interactions between phentermine, dexfenfluramine, and 5-HT2C agonists, on food intake in the rat. Psychopharmacol. (Berl.) 232, 1973–1982. 10.1007/s00213-014-3829-2 PMC4425807 25524140

[B24] Gudelsky G. A. Yamamoto B. K. Frank Nash J. (1994). Potentiation of 3,4-methylenedioxymethamphetamine-induced dopamine release and serotonin neurotoxicity by 5-HT2 receptor agonists. Eur. J. Pharmacol. 264, 325–330. 10.1016/0014-2999(94)90669-6 7698172

[B25] Gutierrez R. Simon S. A. Nicolelis M. A. L. (2010). Licking-induced synchrony in the taste–reward circuit improves cue discrimination during learning. J. Neurosci. 30, 287–303. 10.1523/JNEUROSCI.0855-09.2010 20053910 PMC2831544

[B26] Halford J. C. G. Harrold J. A. Lawton C. L. Blundell J. E. (2005). Serotonin (5-HT) drugs: effects on appetite expression and use for the treatment of obesity. Curr. Drug Targets 6, 201–213. 10.2174/1389450053174550 15777190

[B27] Halladay A. K. Fisher H. Wagner G. C. (2000). Effects of phentermine and fenfluramine on alcohol consumption and alcohol withdrawal seizures in rats. Alcohol 20, 19–29. 10.1016/S0741-8329(99)00047-6 10680713

[B28] Halladay A. K. Wagner G. C. Sekowski A. Rothman R. B. Baumann M. H. Fisher H. (2006). Alterations in alcohol consumption, withdrawal seizures, and monoamine transmission in rats treated with phentermine and 5-hydroxy-L-tryptophan. Synapse 59, 277–289. 10.1002/syn.20239 16416445

[B29] Hampp C. Kang E. M. Borders-Hemphill V. (2013). Use of prescription antiobesity drugs in the United States. Pharmacother. J. Hum. Pharmacol. Drug Ther. 33, 1299–1307. 10.1002/phar.1342 PMC4740913 24019195

[B30] Hendricks E. J. Greenway F. L. Westman E. C. Gupta A. K. (2011). Blood pressure and heart rate effects, weight loss and maintenance during long-term phentermine pharmacotherapy for obesity. Obesity 19, 2351–2360. 10.1038/oby.2011.94 21527891

[B31] Hendricks E. J. Rothman R. B. Greenway F. L. (2009). How physician obesity specialists use drugs to treat obesity. Obesity 17, 1730–1735. 10.1038/oby.2009.69 19300434

[B32] Hendricks E. J. Srisurapanont M. Schmidt S. L. Haggard M. Souter S. Mitchell C. L. (2014). Addiction potential of phentermine prescribed during long-term treatment of obesity. Int. J. Obes. 38, 292–298. 10.1038/ijo.2013.74 23736363

[B33] Hensler J. G. (2006). Serotonergic modulation of the limbic system. Neurosci. Biobehav. Rev. 30, 203–214. 10.1016/j.neubiorev.2005.06.007 16157378

[B34] Higashi H. Inanaga K. Nishi S. Uchimura N. (1989). Enhancement of dopamine actions on rat nucleus accumbens neurones *in vitro* after methamphetamine pre-treatment. J. Physiol. 408, 587–603. 10.1113/jphysiol.1989.sp017478 2550628 PMC1190422

[B35] Hirai M. Nakajima T. (1979). Biochemical studies on the mechanism of difference in the renal toxicity of 5-hydroxy-L-tryptophan between Sprague Dawley and Wistar rats. J. Biochem. (Tokyo) 86, 907–913. 10.1093/oxfordjournals.jbchem.a132622 115856

[B36] Huang X. Nichols D. E. (1993). 5-HT2 receptor-mediated potentiation of dopamine synthesis and central serotonergic deficits. Eur. J. Pharmacol. 238, 291–296. 10.1016/0014-2999(93)90859-G 8104811

[B37] Kalyanasundar B. Perez C. I. Luna A. Solorio J. Moreno M. G. Elias D. (2015). D1 and D2 antagonists reverse the effects of appetite suppressants on weight loss, food intake, locomotion, and rebalance spiking inhibition in the rat NAc shell. J. Neurophysiol. 114, 585–607. 10.1152/jn.00012.2015 25972577 PMC4509405

[B38] Kelley A. E. (2004). Ventral striatal control of appetitive motivation: role in ingestive behavior and reward-related learning. Neurosci. Biobehav. Rev. 27, 765–776. 10.1016/j.neubiorev.2003.11.015 15019426

[B39] Klawonn A. M. Malenka R. C. (2019). Nucleus accumbens modulation in reward and aversion. Cold Spring Harb. Symp. Quant. Biol. 83, 119–129. 10.1101/sqb.2018.83.037457 PMC6650377 30674650

[B40] Lucas J. J. Yamamoto A. Scearce-Levie K. Saudou F. Hen R. (1998). Absence of fenfluramine-induced anorexia and reduced c-fos induction in the hypothalamus and central amygdaloid complex of serotonin 1B receptor knock-out mice. J. Neurosci. 18, 5537–5544. 10.1523/JNEUROSCI.18-14-05537.1998 9651234 PMC6793482

[B41] Marvin J. S. Borghuis B. G. Tian L. Cichon J. Harnett M. T. Akerboom J. (2013). An optimized fluorescent probe for visualizing glutamate neurotransmission. Nat. Methods 10, 162–170. 10.1038/nmeth.2333 23314171 PMC4469972

[B42] McMurray J. Bloomfield P. Miller H. C. (1986). Irreversible pulmonary hypertension after treatment with fenfluramine. Br. Med. J. Clin. Res. Ed. 293, 51–52. 10.1136/bmj.293.6538.51-d PMC1340807 3089403

[B43] Meguid M. M. Fetissov S. O. Varma M. Sato T. Zhang L. Laviano A. (2000). Hypothalamic dopamine and serotonin in the regulation of food intake. Nutrition 16, 843–857. 10.1016/S0899-9007(00)00449-4 11054589

[B44] Mooney M. E. Schmitz J. M. Moeller F. G. Grabowski J. (2007). Safety, tolerability and efficacy of levodopa–carbidopa treatment for cocaine dependence: Two double-blind, randomized, clinical trials. Drug Alcohol Depend. 88, 214–223. 10.1016/j.drugalcdep.2006.10.011 17134849 PMC2693095

[B45] Nautiyal K. M. Hen R. (2017). Serotonin receptors in depression: from A to B. F1000Research 6, 123. 10.12688/f1000research.9736.1 28232871 PMC5302148

[B46] Nicola S. M. Malenka R. C. (1997). Dopamine depresses excitatory and inhibitory synaptic transmission by distinct mechanisms in the nucleus accumbens. J. Neurosci. 17, 5697–5710. 10.1523/JNEUROSCI.17-15-05697.1997 9221769 PMC6573215

[B47] Nowend K. L. Arizzi M. Carlson B. B. Salamone J. D. (2001). D1 or D2 antagonism in nucleus accumbens core or dorsomedial shell suppresses lever pressing for food but leads to compensatory increases in chow consumption. Pharmacol. Biochem. Behav. 69, 373–382. 10.1016/S0091-3057(01)00524-X 11509194

[B48] O’Connor E. C. Kremer Y. Lefort S. Harada M. Pascoli V. Rohner C. (2015). Accumbal D1R neurons projecting to lateral hypothalamus authorize feeding. Neuron 88, 553–564. 10.1016/j.neuron.2015.09.038 26593092

[B49] O’Neil M. J. (2001). The Merck Index- An Encyclopedia of Chemicals, Drugs, and Biologicals- Online. https://www.rsc.org/Merck-Index/monograph/m6156/hydroxytryptophan?q=unauthorize Accessed 4 Oct 2019.

[B50] Patriarchi T. Cho J. R. Merten K. Howe M. W. Marley A. Xiong W.-H. (2018). Ultrafast neuronal imaging of dopamine dynamics with designed genetically encoded sensors. Science 360, eaat4422. 10.1126/science.aat4422 29853555 PMC6287765

[B51] Pennartz C. M. Dolleman-Van der Weel M. J. Kitai S. T. Lopes da Silva F. H. (1992). Presynaptic dopamine D1 receptors attenuate excitatory and inhibitory limbic inputs to the shell region of the rat nucleus accumbens studied *in vitro*. J. Neurophysiol. 67, 1325–1334. 10.1152/jn.1992.67.5.1325 1534574

[B52] Porras G. Matteo V. D. Fracasso C. Lucas G. Deurwaerdère P. D. Caccia S. (2002). 5-HT 2A and 5-HT 2C/2B receptor subtypes modulate dopamine release induced in vivo by amphetamine and morphine in both the rat nucleus accumbens and striatum. Neuropsychopharmacology 26, 311–324. 10.1016/S0893-133X(01)00333-5 11850146

[B53] Pothos E. N. Creese I. Hoebel B. G. (1995). Restricted eating with weight loss selectively decreases extracellular dopamine in the nucleus accumbens and alters dopamine response to amphetamine, morphine, and food intake. J. Neurosci. 15, 6640–6650. 10.1523/JNEUROSCI.15-10-06640.1995 7472425 PMC6578017

[B54] Prado L. Luis-Islas J. Sandoval O. I. Puron L. Gil M. M. Luna A. (2016). Activation of glutamatergic fibers in the anterior NAc shell modulates reward activity in the aNAcSh, the lateral hypothalamus, and medial prefrontal cCortex and transiently stops feeding. J. Neurosci. 36, 12511–12529. 10.1523/JNEUROSCI.1605-16.2016 27974611 PMC6705665

[B55] Roth J. D. Trevaskis J. L. Wilson J. Lei C. Athanacio J. Mack C. (2008). Antiobesity effects of the β-cell hormone amylin in combination with phentermine or sibutramine in diet-induced obese rats. Int. J. Obes. 32, 1201–1210. 10.1038/ijo.2008.91 18560368

[B56] Rothman R. B. (2010). Treatment of obesity with “combination” pharmacotherapy. Am. J. Ther. 17, 596. 10.1097/MJT.0b013e31818e30da 19352140

[B57] Rothman R. B. Baumann M. H. (2006). Balance between dopamine and serotonin release modulates behavioral effects of amphetamine-type drugs. Ann. N. Y. Acad. Sci. 1074, 245–260. 10.1196/annals.1369.064 17105921

[B58] Rothman R. B. Baumann M. H. (2009). Appetite suppressants, cardiac valve disease and combination pharmacotherapy. Am. J. Ther. 16, 354–364. 10.1097/MJT.0b013e31817fde95 19092640 PMC2713386

[B59] Rothman R. B. Blough B. E. Baumann M. H. (2006). Dual dopamine–5-HT releasers: potential treatment agents for cocaine addiction. Trends Pharmacol. Sci. 27, 612–618. 10.1016/j.tips.2006.10.006 17056126

[B60] Rüttimann E. B. Arnold M. Hillebrand J. J. Geary N. Langhans W. (2009). Intrameal hepatic portal and intraperitoneal infusions of glucagon-like peptide-1 reduce spontaneous meal size in the rat *via* different mechanisms. Endocrinology 150, 1174–1181. 10.1210/en.2008-1221 18948395 PMC2654737

[B61] Santamaría A. Arias H. R. (2010). Neurochemical and behavioral effects elicited by bupropion and diethylpropion in rats. Behav. Brain Res. 211, 132–139. 10.1016/j.bbr.2010.03.023 20307582

[B62] Schneider F. Erisson L. Beygi H. Bradbury M. Cohen-Barak O. Grachev I. D. (2018). Pharmacokinetics, metabolism and safety of deuterated L-DOPA (SD-1077)/carbidopa compared to L-DOPA/carbidopa following single oral dose administration in healthy subjects. Br. J. Clin. Pharmacol. 84, 2422–2432. 10.1111/bcp.13702 29959802 PMC6138493

[B63] Smith S. R. Garvey W. T. Greenway F. L. Zhou S. Fain R. Pilson R. (2017). Coadministration of lorcaserin and phentermine for weight management: a 12-week, randomized, pilot safety study. Obesity 25, 857–865. 10.1002/oby.21811 28440045 PMC5518190

[B64] Smith S. R. Weissman N. J. Anderson C. M. Sanchez M. Chuang E. Stubbe S. (2010). Multicenter, placebo-controlled trial of lorcaserin for weight management. N. Engl. J. Med. 363, 245–256. 10.1056/NEJMoa0909809 20647200

[B65] Srivastava G. Apovian C. (2018). Future pharmacotherapy for obesity: new anti-obesity drugs on the horizon. Curr. Obes. Rep. 7, 147–161. 10.1007/s13679-018-0300-4 29504049

[B66] Sternson S. M. Eiselt A.-K. (2017). Three pillars for the neural control of appetite. Annu. Rev. Physiol. 79, 401–423. 10.1146/annurev-physiol-021115-104948 27912679

[B67] Stuber G. D. Wise R. A. (2016). Lateral hypothalamic circuits for feeding and reward. Nat. Neurosci. 19, 198–205. 10.1038/nn.4220 26814589 PMC4927193

[B68] Thomsen W. J. Grottick A. J. Menzaghi F. Reyes-Saldana H. Espitia S. Yuskin D. (2008). Lorcaserin, a novel selective human 5-hydroxytryptamine2C agonist: in vitro and in vivo pharmacological characterization. J. Pharmacol. Exp. Ther. 325, 577–587. 10.1124/jpet.107.133348 18252809

[B69] Turner E. H. Loftis J. M. Blackwell A. D. (2006). Serotonin a la carte: supplementation with the serotonin precursor 5-hydroxytryptophan. Pharmacol. Ther. 109, 325–338. 10.1016/j.pharmthera.2005.06.004 16023217

[B70] Valsamakis G. Konstantakou P. Mastorakos G. (2017). New targets for drug treatment of obesity. Annu. Rev. Pharmacol. Toxicol. 57, 585–605. 10.1146/annurev-pharmtox-010716-104735 28061687

[B71] van Galen K. A. Horst K. W. Booij J. la Fleur S. E. Serlie M. J. (2018). The role of central dopamine and serotonin in human obesity: lessons learned from molecular neuroimaging studies. Metabolism 85, 325–339. 10.1016/j.metabol.2017.09.007 28970033

[B72] Wang G.-J. Volkow N. D. Fowler J. S. (2002). The role of dopamine in motivation for food in humans: implications for obesity. Expert Opin. Ther. Targets 6, 601–609. 10.1517/14728222.6.5.601 12387683

[B73] Wang G.-J. Volkow N. D. Thanos P. K. Fowler J. S. (2004). Similarity between obesity and drug addiction as assessed by neurofunctional imaging. J. Addict. Dis. 23, 39–53. 10.1300/J069v23n03_04 15256343

[B74] Xu P. He Y. Cao X. Valencia-Torres L. Yan X. Saito K. (2017). Activation of serotonin 2C receptors in dopamine neurons inhibits Binge-like Eating in Mice. Biol. Psychiatry 81, 737–747. 10.1016/j.biopsych.2016.06.005 27516377 PMC5148733

[B75] Xu Y. Jones J. E. Kohno D. Williams K. W. Lee C. E. Choi M. J. (2008). 5-HT2CRs expressed by pro-opiomelanocortin neurons regulate energy homeostasis. Neuron 60, 582–589. 10.1016/j.neuron.2008.09.033 19038216 PMC2631191

[B76] Yanovski S. Z. Yanovski J. A. (2014). Long-term drug treatment for obesity: a systematic and clinical review. JAMA 311, 74–86. 10.1001/jama.2013.281361 24231879 PMC3928674

[B77] Zangen A. Nakash R. Overstreet D. H. Yadid G. (2001). Association between depressive behavior and absence of serotonin–dopamine interaction in the nucleus accumbens. Psychopharmacol. (Berl.) 155, 434–439. 10.1007/s002130100746 11441434

