# Supplementary figures and images for: The Triple Combination Phentermine Plus 5-HTP/Carbidopa Leads to Greater Weight Loss, With Fewer Psychomotor Side Effects Than Each Drug Alone
Source: Front Pharmacol. 2019 Nov 6;10:1327. doi: 10.3389/fphar.2019.01327 (PMC6851240; doi:10.3389/fphar.2019.01327)

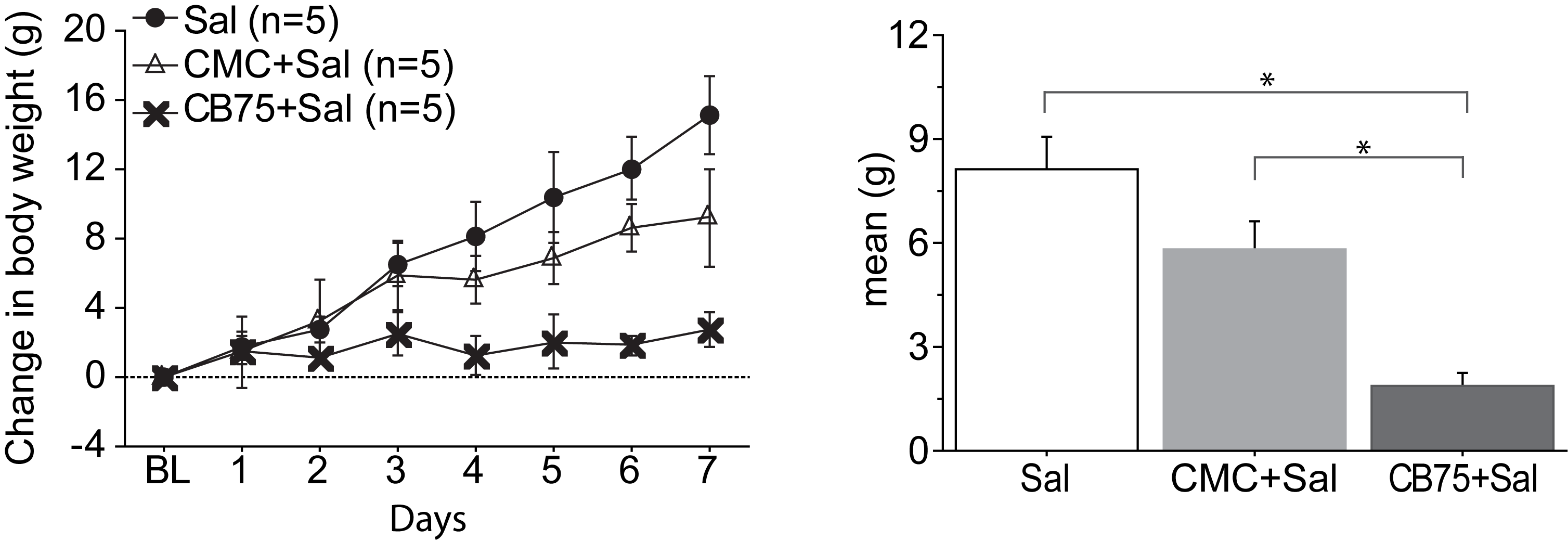

Supplement: Supplementary Figure 1 — Carbidopa alone prevented body weight gain. The left panel depicts the change in body weight across 7 consecutive days of saline (Sal), control carboxymethyl cellulose (CMC+Sal), or carbidopa CB+Sal (75 mg/kg) injections. Rats treated with CB maintained the same body weight relative to their initial weight in BL. The panel at right indicates the average weight change in g. * p< 0.05. Data are mean ± sem. [file Image_1.tif]

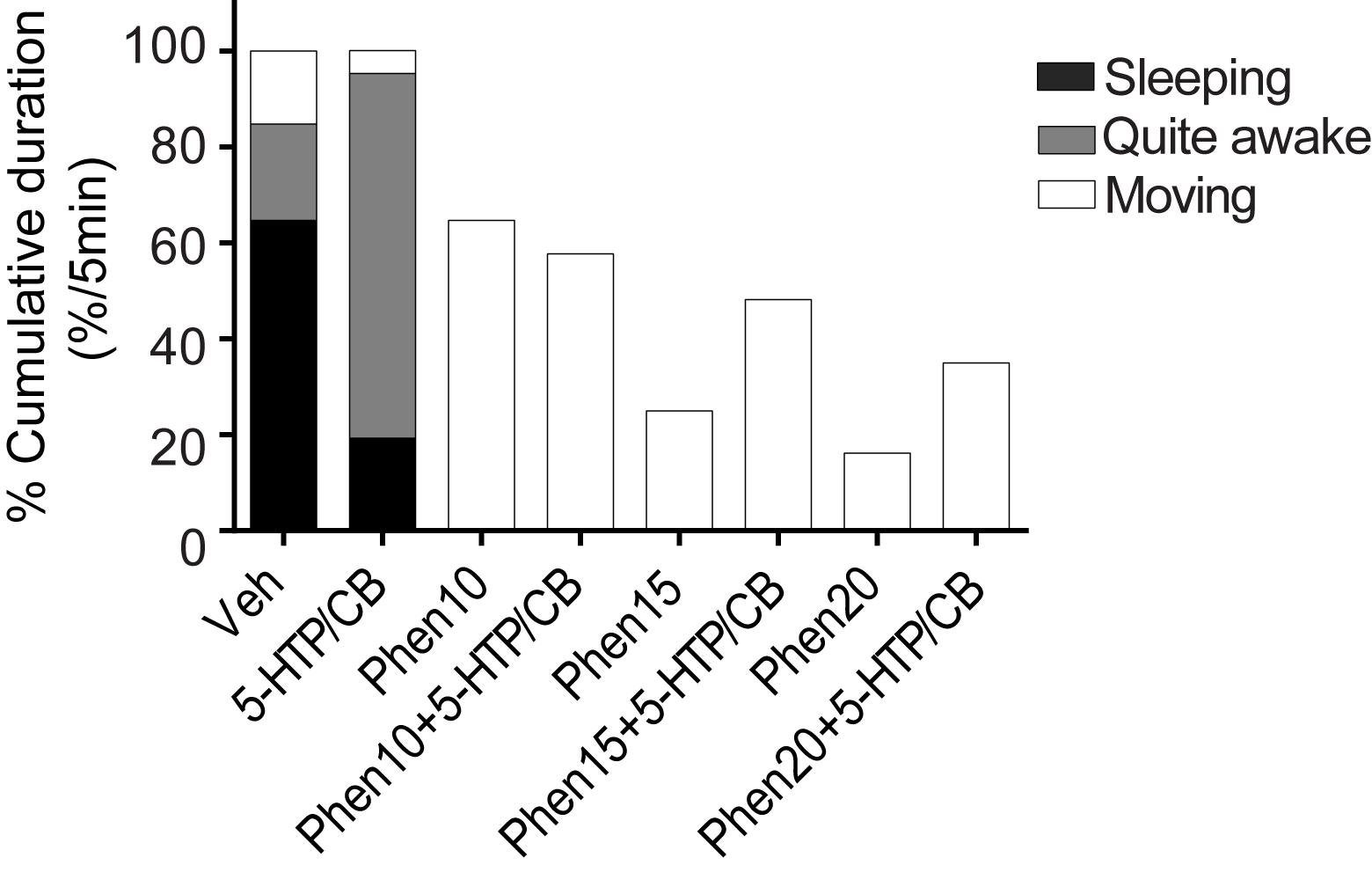

Supplement: Supplementary Figure 2 — Behavioral states induced by appetite suppressants. Percentage of cumulative time spent in each of the following behavioral states: sleeping, quite awake, and moving for 5 min. Measurements were made around 1 h after the onset of drug administration. Data are mean ± sem. [file Image_2.tif]

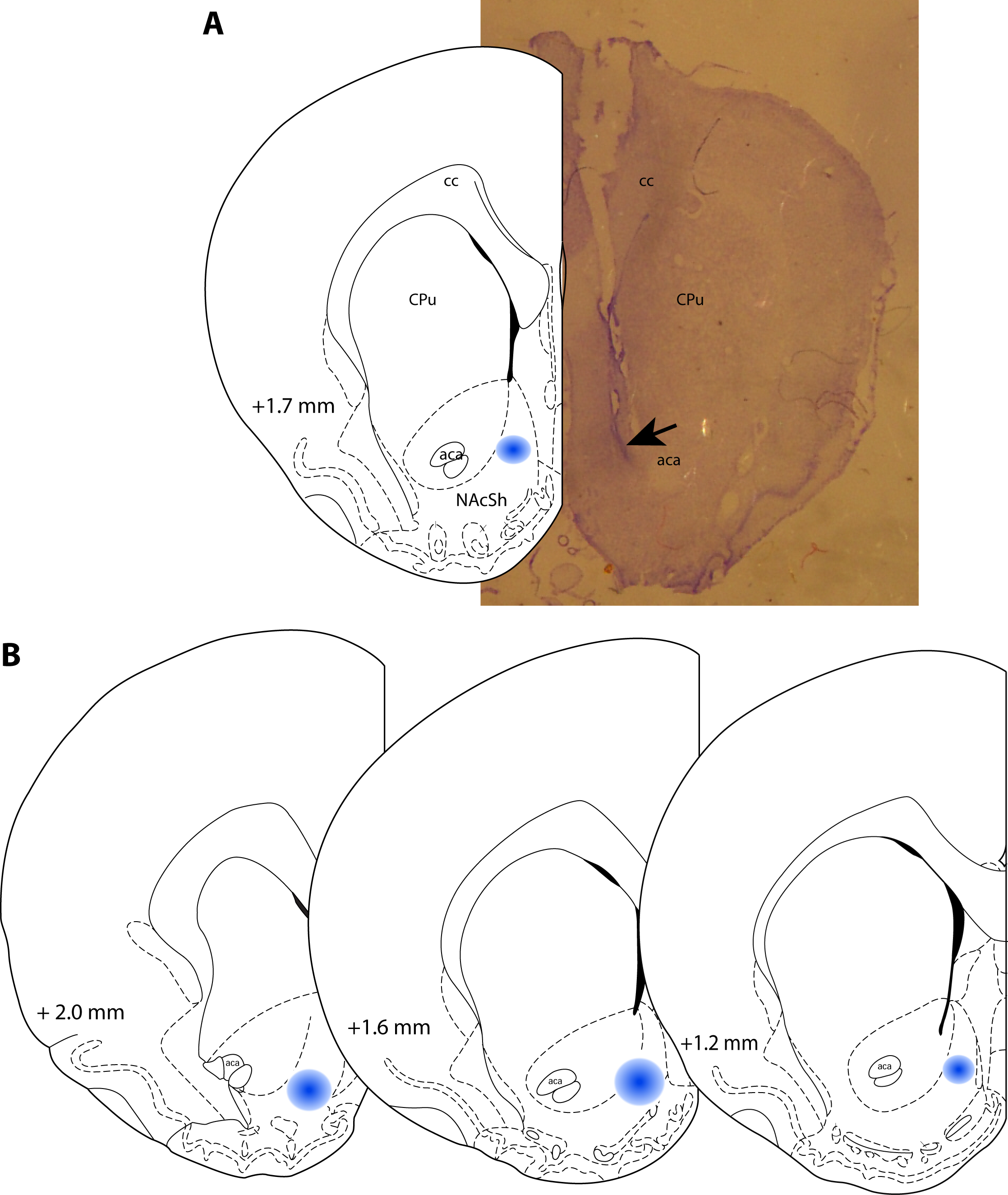

Supplement: Supplementary Figure 3 — Histological reconstruction of electrodes’ tips in NAcSh. A) Nissl-stained coronal section showing an example of a representative electrode track indicated by the black arrow located in NAcSh. B) Summary of NAcSh recording sites across rats. NAcSh, Nucleus Accumbens Shell; cc, Corpus Callosum; aca, Anterior Commissure; CPu, Caudate Putamen (striatum). [file Image_3.tif]
